# Contrasting Modes of New World Arenavirus Neutralization by Immunization-Elicited Monoclonal Antibodies

**DOI:** 10.1128/mbio.02650-21

**Published:** 2022-03-22

**Authors:** Weng M. Ng, Mehmet Sahin, Stefanie A. Krumm, Jeffrey Seow, Antra Zeltina, Karl Harlos, Guido C. Paesen, Daniel D. Pinschewer, Katie J. Doores, Thomas A. Bowden

**Affiliations:** a Division of Structural Biology, Wellcome Centre for Human Genetics, University of Oxford, Oxford, United Kingdom; b Department of Biomedicine, Division of Experimental Virology, University of Basel, Basel, Switzerland; c Kings College London, Department of Infectious Diseases, Guy’s Hospital, London, United Kingdom; Dartmouth College

**Keywords:** arenavirus, glycoprotein, structure, antibody response, monoclonal antibody, antibody-mediated neutralization, host-cell interactions, immunization, structural biology

## Abstract

Transmission of the New World hemorrhagic fever arenaviruses Junín virus (JUNV) and Machupo virus (MACV) to humans is facilitated, in part, by the interaction between the arenavirus GP1 glycoprotein and the human transferrin receptor 1 (hTfR1). We utilize a mouse model of live-attenuated immunization with envelope exchange viruses to isolate neutralizing monoclonal antibodies (NAbs) specific to JUNV GP1 and MACV GP1. Structures of two NAbs, termed JUN1 and MAC1, demonstrate that they neutralize through disruption of hTfR1 recognition. JUN1 utilizes a binding mode common to all characterized infection- and vaccine-elicited JUNV-specific NAbs, which involves mimicking hTfR1 binding through the insertion of a tyrosine into the receptor-binding site. In contrast, MAC1 undergoes a tyrosine-mediated mode of antigen recognition distinct from that used by the reported anti-JUNV NAbs and the only other characterized anti-MACV NAb. These data reveal the varied modes of GP1-specific recognition among New World arenaviruses by the antibody-mediated immune response.

## INTRODUCTION

New World (NW) hemorrhagic fever (HF) arenaviruses are a group of rodent-borne zoonotic pathogens that cause severe disease with high case fatality rates upon transmission into human populations. These agents belong to clades B and D of the genus *Mammarenavirus*, family *Arenaviridae*, and include Junín virus (JUNV), Machupo virus (MACV), Guanarito virus (GTOV), Chapare virus (CHPV), Sabiá virus (SABV), and Whitewater Arroyo virus (WWAV) (1, 2). Although there are currently no internationally approved therapeutic countermeasures available for preventing or treating NW arenaviral HFs, a live attenuated vaccine, Candid#1, has been successfully utilized in Argentina to protect against JUNV and shows evidence of partial cross-protection against MACV (3). Similarly, ribavirin administered intravenously at a high dosage has shown promise in treating Argentine hemorrhagic fever (AHF) (4, 5), and passive immunization with immune plasma containing high titers of neutralizing antibodies (NAbs) from convalescent patients has been shown to be successful in treating JUNV-infected individuals (6, 7).

The mammarenavirus genome comprises single-stranded ambisense bisegmented RNA. The tripartite multifunctional glycoprotein complex (GP) spike that decorates the enveloped virion is encoded within the small (S) RNA segment (8, 9). Each non-covalently associated protomer of the trimeric GP consists of a myristoylated stable signal peptide (SSP) (10, 11), a GP1 receptor-binding glycoprotein (12, 13), and a membrane-anchored GP2 fusion glycoprotein (14–16). Entry of NW clade B and D arenaviruses into a host cell is initiated by the specific interaction of the GP with host cell receptors, including C-type lectins (17, 18), TIMS (T cell/transmembrane, immunoglobulin, and mucin) (18), and the transferrin receptor 1 (TfR1) of their corresponding rodent host (19–22). The ability of NW arenaviruses to also utilize human TfR1 (hTfR1) supposedly is a prerequisite of zoonosis and pathogenicity in humans (20, 23). Following cellular receptor attachment, virions are internalized via endocytosis and delivered to acidified endosomes (9). The low-pH environment that accompanies this process promotes release of GP1 and structural rearrangements of GP2, which fuse viral and host cell membranes, allowing release of the viral genome into the host cell (24, 25).

TfR1 is a homodimeric type II transmembrane protein, where each monomer is composed of a cytoplasmic subunit, a single-pass transmembrane region, and an extracellular domain (ECD). The ECD is further subdivided into three subdomains: a membrane-proximal protease-like domain, a central helical domain that facilitates dimerization, and a membrane-distal apical domain (26). The GP1 subunit contacts the apical domain of TfR1, away from transferrin and hereditary hemochromatosis protein recognition sites (12, 23). The GP1-TfR1 interaction involves the insertion of a tyrosine residue (Tyr211 in hTfR1) into the central pocket on the GP1 surface (23). Structural determination of antigen-binding fragments (Fab) from several NAbs (OD01 [27], GD01 [28], and CR1-28 [29]) of different germ line origins bound to JUNV GP1 revealed a similar mode of recognition, establishing receptor mimicry as a common means of antibody-mediated neutralization. However, this mode of antibody recognition was not observed in the structure of the Fab fragment from cross-reactive MACV/JUNV NAb (CR1-07) in complex with MACV GP1 (29), and there is currently no reported structure of a MACV GP1-specific antibody.

The generation of NAbs has been shown to be effective for the prevention and treatment of HF arenaviral infection (6, 7, 30). Here, we utilized a mouse model of MACV and JUNV live-attenuated immunization combined with antigen-specific B cell sorting to isolate a NAb specific to MACV GP1 (termed MAC1) and seven NAbs specific to JUNV GP1 (termed JUN1 to -7), which recognize an overlapping epitope. Crystallographic investigation of the Fab fragments of NAb MAC1 and a representative JUNV-specific NAb, JUN1, in complex with their cognate GP1 antigens reveals that both NAbs target the TfR1 binding site. Close examination of the antigen-antibody interface reveals that while NAb JUN1 originates from a unique germ line, it utilizes a mode of antigen recognition involving TfR1 mimicry common to all characterized infection- and vaccine-elicited JUNV-specific NAbs. In contrast, NAb MAC1 undergoes a mode of antigen recognition that is distinct from that utilized by reported anti-JUNV NAbs and the cross-reactive MACV/JUNV NAb. Combined, these molecular-level findings expand our appreciation of the varied means by which the antibody-mediated immune response can target the GP1 of NW HF arenaviruses.

## RESULTS

### Isolation and *in vitro* characterization of JUNV and MACV GP1-specific MAbs.

**FIG 1:**
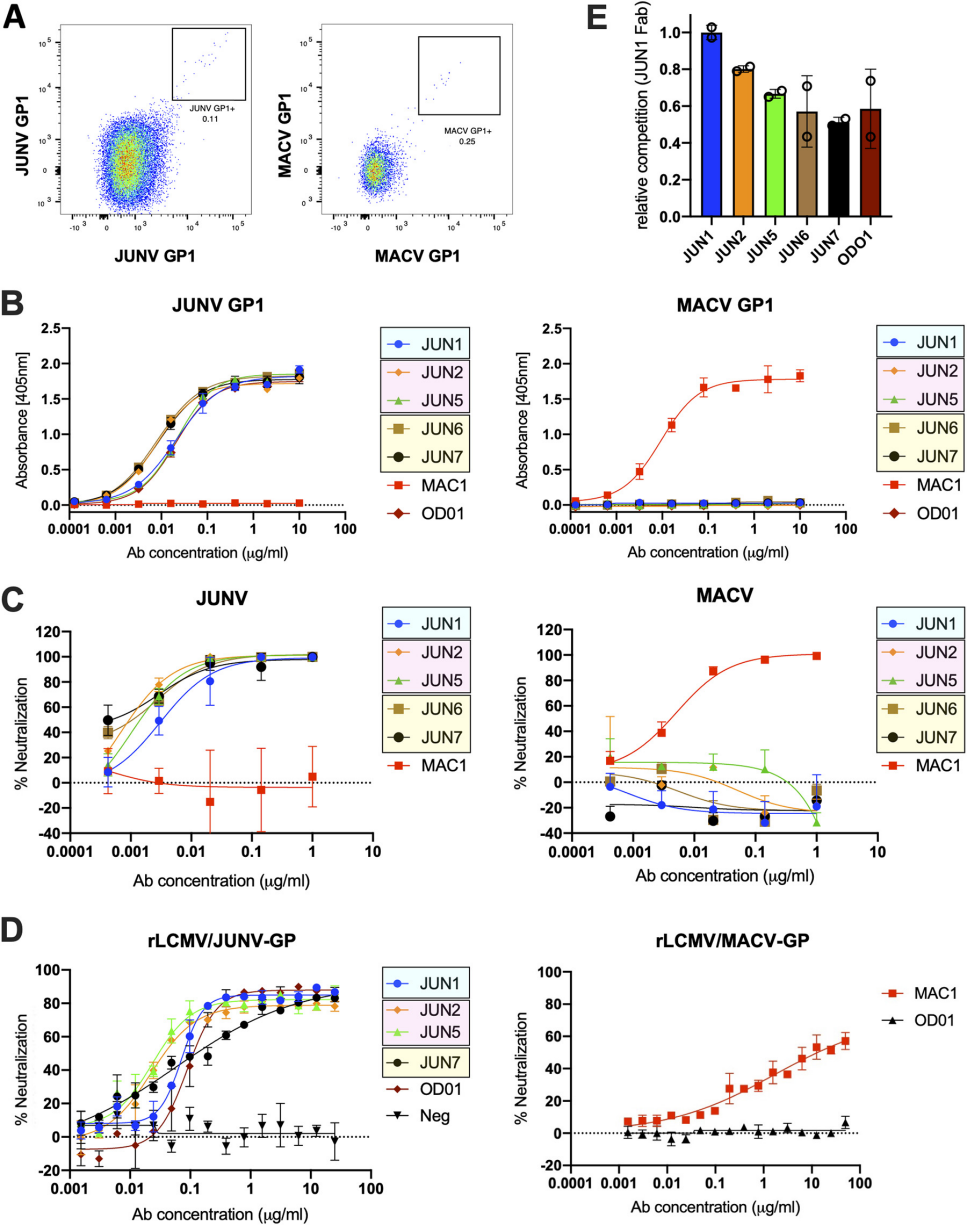
Isolation and characterization of JUNV and MACV GP1-reactive MAbs. (A) Antigen-specific B cell sorting was used to isolate JUNV and MACV GP1-specific NAbs. Figure shows CD4/CD8^−^ B220^+^ IgM^−^ IgD^−^ cells that are either JUNV GP1 or MACV GP1 specific. (B) Binding of MAbs JUN1 to -7 and MAC1 to JUNV and MACV GP1 by ELISA. Colored boxes indicate sequence-related MAbs. A JUNV GP1-specific NAb, OD01 (33), was used as a positive (left panel) and negative (right panel) control. (C) Neutralization by MAbs JUN1 to -7 and MAC1 against HIV-1-based virus particles, pseudotyped with full-length JUNV GP and MACV GP. (D) Neutralization of rLCMV/JUNV-GP and rLCMV/MACV-GP chimeric viruses by JUN1 to -7 and MAC1 was tested in an immunofocus reduction neutralization test. OD01 (33) and a non-neutralizing JUNV GP1-specific MAb (denoted as Neg) isolated as a part of this study were included as the positive and negative controls, respectively, in the rLCMV/JUNV-GP neutralization test by JUN1 to -7. (E) Competition between JUNV NAbs and JUN1 Fab for binding to JUNV GP1 determined by ELISA. Competition is reported relative to the maximum competition observed between JUN1 Fab and JUN1 IgG. The assays presented in panels B to E were performed in duplicate and repeated at least twice. Representative plots are shown. Error bars represent the range of the value for experiments performed in duplicate (not shown when smaller than symbol size).

To interrogate the antibody-mediated immune response to live-attenuated arenavirus immunization, we performed antigen-specific B cell sorting of splenocytes derived from mice vaccinated with Candid#1 and with genetically engineered biosafety level 2 (BSL-2) arenaviruses (31, 32) expressing as their envelope protein the GPs of either JUNV or MACV, respectively (Fig. 1A). We recovered the antibody sequences and recombinantly produced seven JUNV GP1-specific monoclonal antibodies (MAbs) and one MACV GP1-specific MAb. JUNV-neutralizing MAbs were derived from a mouse immunized with three doses of recombinant lymphocytic choriomeningitis virus (rLCMV)/JUNV-GP and a final dose of Candid#1. For the isolation of the MACV-neutralizing antibody MAC1, and in an effort to derive cross-reactive JUNV/MACV-GP NAbs, mice were primed with rLCMV/MACV-GP and boosted with recombinant Pichinde virus (rPICV)/JUNV-GP and rPICV/MACV-GP. All JUNV GP1 and MACV GP1-directed MAbs exhibited a high level of binding and were singly specific to JUNV GP1 and MACV GP1, respectively, with apparent affinities on par with that exhibited by the JUNV GP1-specific NAb, OD01 (27, 33) (Fig. 1B). The seven anti-JUNV GP1 MAbs, termed JUN1 to -7, and one anti-MACV MAb, termed MAC1, originate from different germ lines than those previously identified for anti-NW GP1 NAbs (see Tables S1 and S2 and Fig. S1 in the supplemental material). With the exception of NAb JUN1, the heavy chain of each of the JUNV NAbs likely originated from the IGHV1-52*01 germ line, with NAb JUN2 to -5 light chains originating from the IGKV14-111*01 kappa germ line, and NAb JUN6 and -7 light chains originating from the IGKV3-2*01 kappa germ line. In total, this allowed us to identify three genetically distinct lineages of JUNV GP1-specific MAbs (Table S2).

10.1128/mBio.02650-21.1 FIG S1 Amino acid sequence alignments of Fab JUN1 and Fab MAC1 with respect to their closest corresponding germ line sequences. Alignments of the variable domains from the JUN1 heavy chain (A), JUN1 light chain (B), MAC1 heavy chain (C), and MAC1 light chain (D) are shown. Top germ line V-gene hits were determined using IMGT, the international ImMunoGeneTics information system (http://www.imgt.org) (52). Sequence alignments were prepared using MultAlin (67), plotted with ESPRIPT (68), and adjusted by hand. Amino acid numbers corresponding to JUN1 CDR H3 Tyr100A and MAC1 CDR H3 Tyr97 are highlighted with red boxes. HFR and LFR denote heavy and light chain framework, respectively. Download FIG S1, TIF file, 2.6 MB. Copyright © 2022 Ng et al. 2022 Ng et al. https://creativecommons.org/licenses/by/4.0/ This content is distributed under the terms of the Creative Commons Attribution 4.0 International license.

10.1128/mBio.02650-21.7 TABLE S1 Summary for structurally characterized anti-JUNV and anti-MACV NAbs. Download Table S1, DOCX file, 0.02 MB. Copyright © 2022 Ng et al. 2022 Ng et al. https://creativecommons.org/licenses/by/4.0/ This content is distributed under the terms of the Creative Commons Attribution 4.0 International license.

10.1128/mBio.02650-21.8 TABLE S2 Summary of anti-JUNV and anti-MACV NAb heavy and light chain features. Download Table S2, DOCX file, 0.02 MB. Copyright © 2022 Ng et al. 2022 Ng et al. https://creativecommons.org/licenses/by/4.0/ This content is distributed under the terms of the Creative Commons Attribution 4.0 International license.

JUN1 to -7 (50% inhibitory concentration [IC_50_] 0.001 to 0.003 μg/mL) and MAC1 (IC_50_ 0.004 μg/mL) exhibited neutralizing potency against HIV-1 (human immunodeficiency virus type 1)-based virus particles, pseudotyped with full-length JUNV GP and MACV GP, respectively (Fig. 1C). These NAbs also neutralize (IC_50_ 0.030 to 0.125 μg/mL for JUN1 to -7 and 13.0 μg/mL for MAC1) in an immunofocus reduction neutralization assay, which is based on the envelope-chimeric LCMV used for live-attenuated immunization (Fig. 1D) (31). The observed LCMV-based assay IC_50_ values are similar to those observed for the previously reported NAbs, including structurally characterized OD01, GD01, and CR1-28 (28, 29, 31, 34). Although a heterologous prime-boost immunization was performed in an effort to derive cross-reactive JUNV/MACV NAbs, none of the isolated MAbs exhibited cross-neutralizing potency *in vitro* (Fig. 1C). To assess whether the three anti-JUNV GP1 NAb groups compete for the same region of the GP1, we performed an enzyme-linked immunosorbent assay (ELISA)-based competition assay (Fig. 1E), which suggests that each of the identified anti-JUNV GP1 NAb groups recognizes overlapping epitopes on JUNV GP1.

### JUNV GP1-directed NAb JUN1 utilizes receptor mimicry for antigen recognition and virus neutralization.

Given that JUN1 to -7 all target a proximally similar epitope and display neutralization potency within the same order of magnitude (Fig. 1C), we used NAb JUN1 as a representative from the isolated MAbs to determine the structural basis for neutralization. Recombinant JUNV GP1 was complexed with the Fab fragment of NAb JUN1, partially deglycosylated with endoglycosidase F1 (endoF1), and crystallized using the sitting drop vapor diffusion method. X-ray diffraction data were collected to 2.5-Å resolution (Table S3), and the structure was solved by molecular replacement using structures of JUNV GP1 and Fab OD01 (PDB ID: 5NUZ [27]) as search models. One JUNV GP1-Fab JUN1 complex was present in the asymmetric unit.

10.1128/mBio.02650-21.9 TABLE S3 Crystallographic data collection and refinement statistics. Download Table S3, DOCX file, 0.02 MB. Copyright © 2022 Ng et al. 2022 Ng et al. https://creativecommons.org/licenses/by/4.0/ This content is distributed under the terms of the Creative Commons Attribution 4.0 International license.

**FIG 2:**
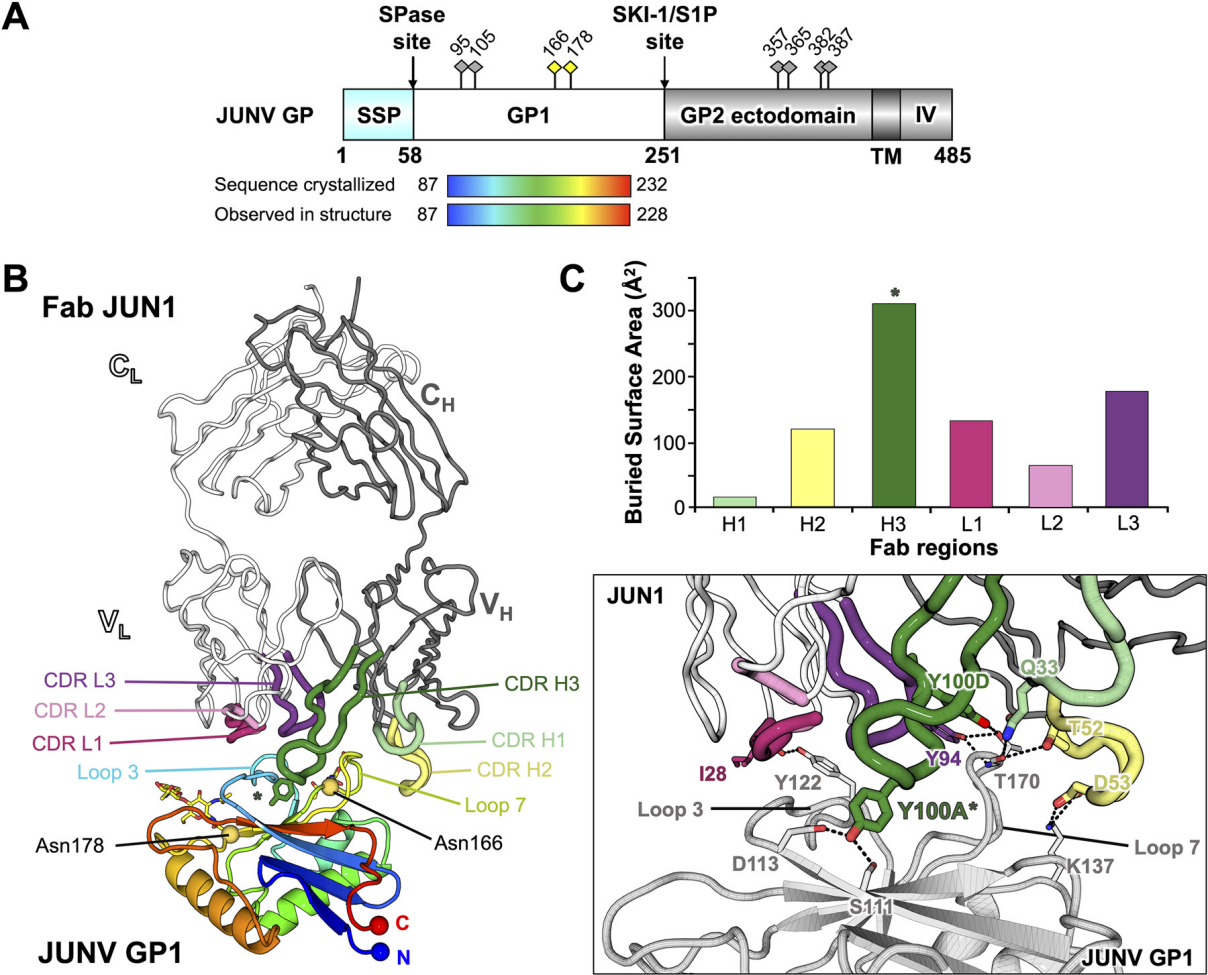
Structural basis for Fab JUN1 recognition of JUNV GP1. (A) Schematic domain organization of the JUNV GP (generated with DOG software [65]). The JUNV GP1 construct used for crystallization is highlighted as a rainbow. The stable signal peptide (SSP), signal peptidase (SPase) site, GP1 glycoprotein, subtilisin-like kexin protease 1-site 1 protease (SKI-1/S1P) cleavage site, GP2 glycoprotein, transmembrane region (TM), and intravirion domain (IV) are annotated. Putative N-linked glycosylation sites are labeled as diamond-shaped pins above the schematic, with sites observed in the crystal structure colored yellow. (B) Structure of JUNV GP1 in complex with Fab JUN1. JUNV GP1 is shown in cartoon representation and colored as a rainbow ramped from blue (N terminus) to red (C terminus). Crystallographically observed N-linked glycosylation sites are labeled and indicated as yellow spheres with the glycans shown as sticks. Fab JUN1 is shown in ribbon representation, with heavy chain colored gray and light chain colored white. CDR loops contributing to JUNV GP1 recognition are labeled and colored light green (CDR H1), yellow (CDR H2), dark green (CDR H3), pink (CDR L1), light pink (CDR L2), and purple (CDR L3). The side chain of Tyr100A is shown as a stick and denoted with an asterisk. V_H_, V_L_, C_H_, and C_L_ denote the antibody variable heavy, variable light, constant heavy, and constant light chain domains, respectively. (C) Interaction between JUNV GP1 and Fab JUN1. (Upper panel) A bar chart representing the contributions of each JUN1 CDR toward JUNV GP1 binding, calculated using the PDBePISA server (66) and measured in buried surface area (Å^2^). The bar corresponding to the CDR loop carrying Tyr100A is denoted with an asterisk. (Lower panel) Closeup view of the JUNV GP1-Fab JUN1 interface. JUNV GP1 is colored light gray, and Fab JUN1 is colored as in panel B. Selected intermolecular hydrogen bonds are highlighted with dashes, and participating residues are shown as sticks.

**FIG 3:**
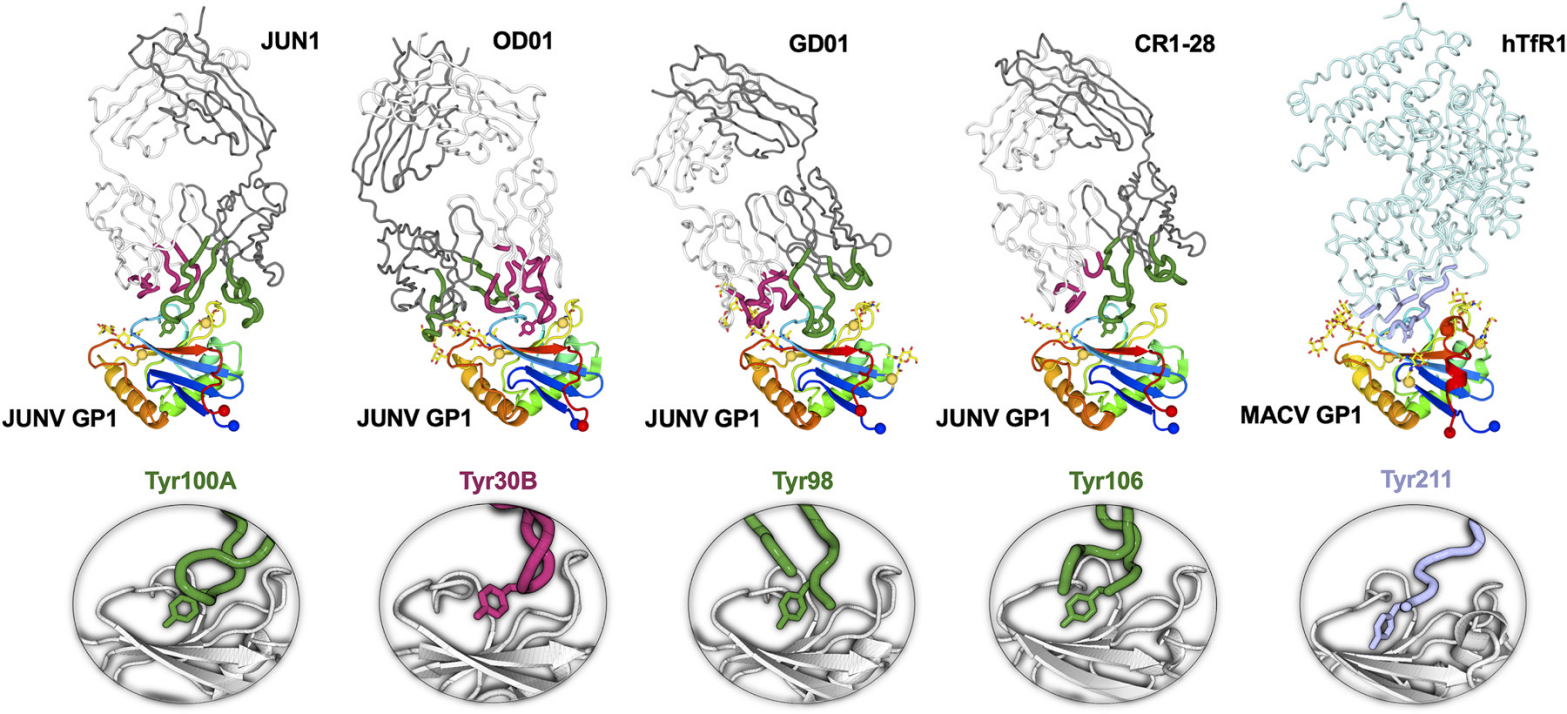
Tyrosine-mediated JUNV GP1 and MACV GP1 recognition: a common mode of MAb neutralization. (Upper row) JUNV GP1 and MACV GP1 in complex with Fab fragments of NAbs (JUN1, OD01 [27], GD01 [28], and CR1-28 [29]) and receptor (hTfR1 [23]), respectively. GP1 is shown in cartoon representation and colored as a rainbow from blue (N terminus) to red (C terminus). N-linked glycosylation sites are indicated as yellow spheres with glycans shown as sticks. Heavy and light chains of Fab fragments are shown as gray and white ribbons, respectively. CDRs contributing to JUNV recognition are colored dark green (heavy chain) and pink (light chain). HTfR1 is shown as a light blue ribbon with regions that interact with MACV shown as blue, thicker ribbons. (Lower row) Closeup view of the key tyrosine residues (sticks) that are inserted into the central pocket of the GP1 (light gray cartoon). Only parts of CDR loops containing the tyrosine residues are shown. CDR residues hindering the view of tyrosine are removed for clarity.

As previously observed (27–29), JUNV GP1 (Asp87 to Asn232) forms a compact α/β fold consisting of a central seven-stranded β-sheet that constitutes the receptor binding site (RBS) on one face and three α-helices on the opposing face (Fig. 2A and B). Superposition analysis demonstrates that our JUNV GP1 exhibits a high level of structural conservation when superimposed with the structures of JUNV GP1 in previously reported Fab-bound states (an average of 0.6-Å root mean square deviation [RMSD] over equivalent Cα residues). Fab JUN1 binds to JUNV GP1 at a site overlapping that used for TfR1 recognition through the use of all six complementarity-determining regions (CDRs) (Fig. 2B and C), in an extensive interaction that occludes approximately 1,550 Å^2^ of solvent-accessible surface area. Although the CDRs of both the heavy and light chains of Fab JUN1 make a similar contribution to the overall amount of surface area occluded in the GP1-JUN1 interface, the CDR3 of the heavy chain (CDR H3) is central to the interaction and extends a 16-amino-acid loop into a central pocket formed by the β-sheet and loop 3 of JUNV GP1 (Fig. 2B and C). Within the CDR H3 loop, the side chain of Tyr100A (Chothia numbering scheme [35]) inserts into the pocket and interacts with Ser111 and Asp113 at the tip of the third β-strand of the JUNV GP1 (Fig. 2C). Interestingly, despite originating from a unique germ line (Table S1; Fig. S1A and B), Fab JUN1 employs a tyrosine-mediated mode of antigen recognition that is common to that utilized by previously characterized NAbs, namely, OD01 (27), GD01 (28), and CR1-28 (29). Indeed, the insertion of Tyr100A from NAb JUN1 into the central pocket of JUNV GP1 is mirrored by Tyr30B of the NAb OD01 light chain and Tyr98 and Tyr106 of NAb GD01 and NAb CR1-28 heavy chains, respectively (Fig. 3). Moreover, this mode of tyrosine-mediated GP1 engagement is highly reminiscent of the insertion of Tyr211 from hTfR1 in the MACV GP1-hTfR1 complex, the only structurally characterized NW GP1-host receptor complex to date (23) (Fig. 3). In line with previous comparisons of JUNV GP1-bound NAb structures, the identification of this antigen-antibody recognition mode by an infection-elicited NAb confirms TfR1 receptor mimicry as a potent and common means of antibody-mediated neutralization of JUNV.

Although the insertion of Tyr100A from Fab JUN1 into the pocket of JUNV GP1 represents the signature feature of the complex, the antigen-antibody binding interface also involves secondary interactions between loops 3 and 7 of JUNV GP1 and CDR H2, H3, L1, and L3 of Fab JUN1 (Fig. 2C; Fig. S2). Indeed, an extensive secondary interface is supported by a network of hydrogen bonds between Thr170^JUNV GP1^ and Glu171^JUNV GP1^ from loop 7 and Gln33^CDR H1^, Asn58^CDR H2^, Tyr100D^CDR H3^, and Tyr94^CDR L3^ of Fab JUN1 (Fig. S2). Residues Gln27^CDR L1^ to Ser32^CDR L1^ also provide substantial contacts with loop 3 of JUNV GP1, including hydrogen bonding between Ile28^CDR L1^ and Tyr122^JUNV GP1^ (Fig. S2). The inclusion of these further contacts is, in part, facilitated by the angle by which Fab JUN1 approaches the GP1 RBS. Indeed, this mode of engagement is most similar to that of Fab CR1-28 (an approximately 10° deviation in the relative angle of approach), with respect to Fabs GD01 and OD01 (deviations of approximately 20° and 25°, respectively) (Fig. 3).

10.1128/mBio.02650-21.2 FIG S2 Schematic diagram of contacts formed in the JUNV GP1-Fab JUN1 interface. (Top) GP1-JUN1 heavy chain CDR interactions. (Bottom) GP1-JUN1 light chain CDR interactions. JUNV GP1 residues are colored gray, and Fab JUN1 residues are colored green. Atoms corresponding to carbon, nitrogen, and oxygen are shown as black, blue, and red balls, respectively. Residues involved in hydrogen bonding and salt bridges are shown as sticks; residues involved in hydrophobic interactions are shown as spoked arcs. Hydrogen bonds/salt bridges and hydrophobic interactions are shown as cyan and green dotted lines, respectively. Plots were generated with LigPlot+ (69). Download FIG S2, TIF file, 1.7 MB. Copyright © 2022 Ng et al. 2022 Ng et al. https://creativecommons.org/licenses/by/4.0/ This content is distributed under the terms of the Creative Commons Attribution 4.0 International license.

Four N-linked glycosylation sequons encompass the periphery of the JUNV GP1 RBS (Fig. 2A and B): Asn95, Asn105, Asn166, and Asn178. Electron density corresponding to at least one well-ordered GlcNAc moiety was observed at Asn166 and Asn178 but not at Asn95 and Asn105 in the structure of JUNV GP1-Fab JUN1 (Fig. 2B; Fig. S4A). Given that N-linked glycosylation was observed at Asn105 in a previously crystallized JUNV GP1 molecule produced under similar conditions (PDB ID 5NUZ [27]), it is likely that the glycan extending from this site is intrinsically flexible in the crystal. The absence of structural evidence for the glycan at Asn95 in this or any other reported JUNV GP1 structures suggests that this site may not be glycosylated to sufficient occupancy during protein folding, at least under the recombinant expression conditions used for protein production. Examination of the JUNV GP1-Fab JUN1 interface indicates that the epitope is protein specific and that glycosylation is unlikely to play a role in supporting the interaction (Fig. 2C). However, the close proximity of these glycans to the JUN1 epitope is consistent with their established role in shielding the GP surface and reducing neutralizing potential, as has been observed for other anti-JUNV GP1 RBS NAbs, including GD01 and OD01 (31).

10.1128/mBio.02650-21.4 FIG S4 Visualization of N-linked glycans on JUNV and MACV GP1. JUNV GP1 (A) and MACV GP1 (B) are shown in cartoon representation and colored light gray. N-linked glycans are shown as sticks with atoms corresponding to carbon, nitrogen, and oxygen colored yellow, blue, and red, respectively. Electron density (composite omit map contoured at 1.0 σ, blue) is shown for ordered N-linked glycans observed in the crystal structures of JUNV GP1-Fab JUN1 and MACV GP1-Fab MAC1. Closeup view of each N-linked glycan and its density are shown and annotated, including a chain of Man_7_GlcNAc_2_ on Asn178^MACV GP1^ that interacts with residues at the C-terminal region of the Fab MAC1 CDR L2. Intermolecular hydrogen bonds are highlighted with dashes, and participating residues are shown as sticks. Fab MAC1 is shown as a white ribbon with CDR L2 in pink. Download FIG S4, TIF file, 2.5 MB. Copyright © 2022 Ng et al. 2022 Ng et al. https://creativecommons.org/licenses/by/4.0/ This content is distributed under the terms of the Creative Commons Attribution 4.0 International license.

### Structural basis for MACV GP1 recognition by NAb MAC1.

MAC1 is currently the only NAb with a known sequence that is solely specific to and neutralizes MACV (Table S1; Fig. S1C and D). To characterize the mode of MAC1 recognition, we crystallized MACV GP1 in complex with the Fab fragment of NAb MAC1 using a similar strategy as that described for the JUNV GP1-Fab JUN1 complex. Briefly, following deglycosylation, complex formation, and crystallization, X-ray diffraction data were collected to 1.9-Å resolution (Table S3), and the structure was solved by molecular replacement using MACV GP1 (PDB ID: 2WFO [13]) and Fab OD01 (PDB ID: 5NUZ [27]) structures as search models. One copy of the antigen-Fab MAC1 complex was present in the asymmetric unit.

**FIG 4:**
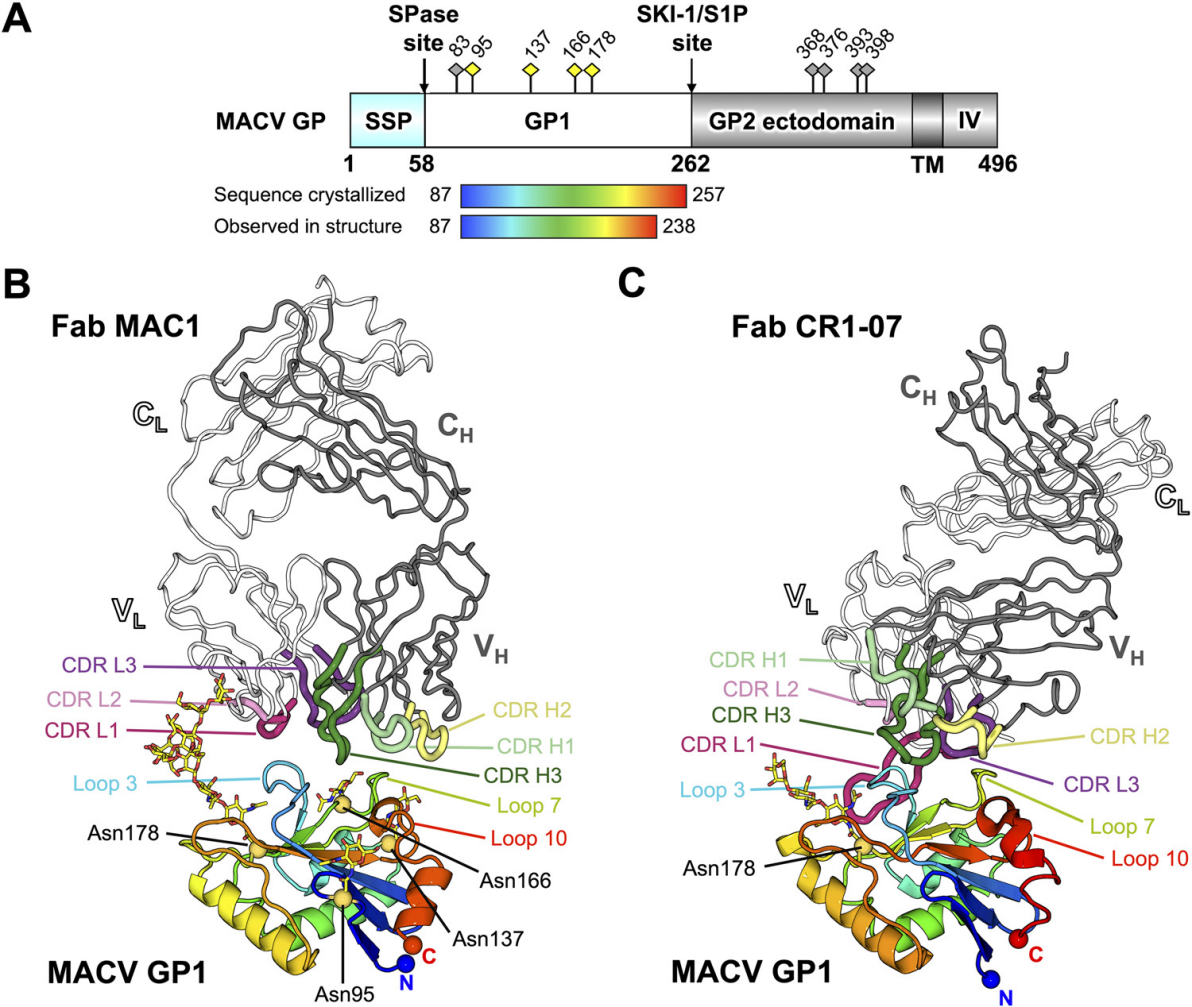
Structural basis for Fab MAC1 recognition of MACV GP1. (A) Schematic domain organization of the MACV GP (generated with DOG software [65]). The MACV GP1 construct used for crystallization is highlighted as a rainbow. The stable signal peptide (SSP), signal peptidase (SPase) site, GP1 glycoprotein, subtilisin-like kexin protease 1-site 1 protease (SKI-1/S1P) cleavage site, GP2 glycoprotein, transmembrane region (TM), and intravirion domain (IV) are annotated. Putative N-linked glycosylation sites are labeled as diamond-shaped pins above the schematic, with sites observed in the crystal structure colored yellow. (B) Structure of MACV GP1 in complex with Fab MAC1. MACV GP1 is shown in cartoon representation and colored as a rainbow ramped from blue (N terminus) to red (C terminus). Crystallographically observed N-linked glycosylation sites are labeled and indicated as yellow spheres with the glycans shown as sticks. MAC1 is shown as in ribbon representation, with heavy chain colored gray and light chain colored white. CDR loops are labeled and colored light green (CDR H1), yellow (CDR H2), dark green (CDR H3), pink (CDR L1), light pink (CDR L2), and purple (CDR L3). V_H_, V_L_, C_H_, and C_L_ denote the antibody variable heavy, variable light, constant heavy, and constant light chain domains, respectively. (C) Structure of MACV GP1 in complex with Fab CR1-07 (PDB ID: 5W1M) (29). The structure is presented, colored, and annotated as in panel B.

**FIG 5:**
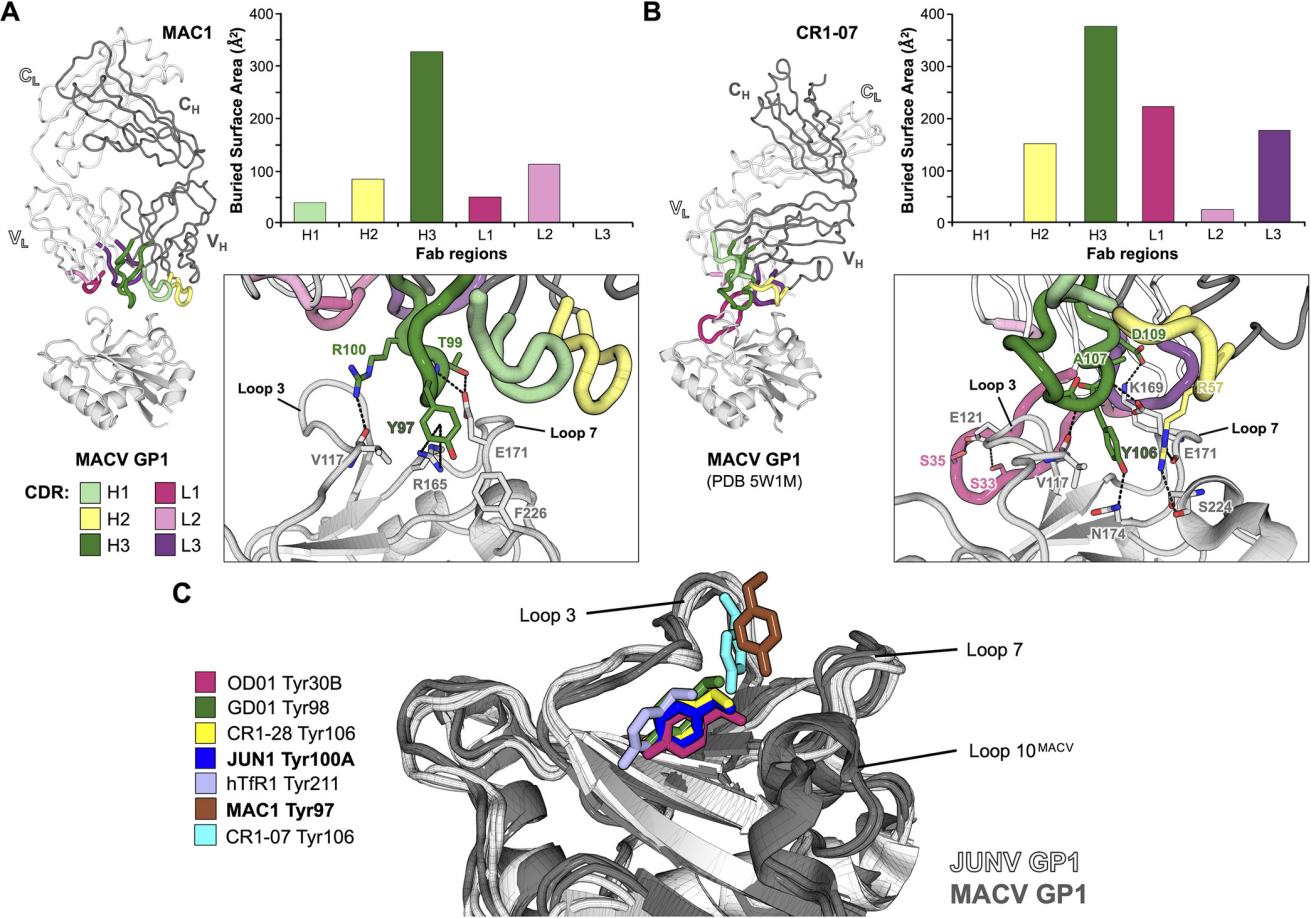
Comparison of MACV GP1-Fab MAC1 and MACV GP1-Fab CR1-07 complex interfaces. (A) Interaction between MACV GP1 and Fab MAC1. (Left) MACV GP1 is shown as a light gray cartoon. Fab MAC1 is shown as dark gray (heavy chain) and white (light chain) ribbons, with CDR loops colored as indicated. (Upper right) A bar chart representing the contributions of each MAC1 CDR toward MACV GP1 binding, calculated using the PDBePISA server (66) and measured in buried surface area (Å^2^). (Lower right) Closeup view of the MACV GP1-Fab MAC1 interface. Selected intermolecular hydrogen bonds are highlighted with dashes, and participating residues are shown as sticks. (B) Interaction between MACV GP1 and Fab CR1-07. (Left) Structure of MACV GP1-Fab CR1-07 (PDB ID: 5W1M) (29), presented as in panel A. (Upper right) A bar chart representing the contributions of each CR1-07 CDR toward MACV GP1 binding, calculated as in panel A. (Lower right) Closeup view of the MACV GP1-Fab CR1-07 interface, presented as in panel A. (C) Overlay of signature CDR tyrosine residues (stick representation) from OD01 (Tyr30B, pink), GD01 (Tyr98, dark green), CR1-28 (Tyr106, yellow), JUN1 (Tyr100A, dark blue), hTfR1 (Tyr211, blue), MAC1 (Tyr97, brown), and CR1-07 (Tyr106, cyan) with respect to JUNV GP1 (white cartoon) and MACV GP1 (gray cartoon).

As previously observed (13), MACV GP1 (Glu87 to Phe257) adopts the expected compact α/β fold (Fig. 4A and B) and exhibits a high level of structural conservation when superimposed with the unliganded form of MACV GP1 (0.7-Å RMSD; PDB ID 2WFO [13]), as well as MACV GP1 bound to hTfR1 (0.7-Å RMSD; PDB ID 3KAS [23]) and the MACV/JUNV cross-reactive Fab, CR1-07 (0.9-Å RMSD; PDB ID 5W1M [29]) (Fig. 4C). Fab MAC1 interacts with the cognate GP1 molecule at the structurally observed hTfR1 binding site in an interaction that occludes ∼1,230 Å^2^ of solvent-accessible surface area. Five of the six Fab MAC1 CDRs are involved in MACV GP1 recognition, where the heavy chain contributes to the bulk of the interaction (Fig. 5A).

While characterized JUNV-specific NAbs mimic the native hTfR1-GP1 interaction, namely, the insertion of Tyr211^hTfR1^ into a pocket of MACV GP1 (23) (Fig. 3), this mode of recognition was not recapitulated in the MACV GP1-Fab MAC1 structure. Indeed, the central TfR1 binding pocket remains unoccupied, and in contrast to the long CDR loops that dominate JUNV GP1-NAb contacts, the 13-amino-acid MAC1 CDR H3 sits shallowly and approximately equidistant between loops 3 and 7 of MACV GP1 (Fig. 4B and Fig. 5A). The CDR H3 of Fab MAC1 presents a tyrosine (Tyr97^CDR H3^ [Chothia numbering scheme {35}]), which points toward the RBS pocket (Fig. 5A). However, unlike Tyr211^hTfR1^, which engages Ser113^MACV GP1^ within the RBS pocket (23), Tyr97^CDR H3^ from Fab MAC1 interacts with Val117^MACV GP1^, Arg165^MACV GP1^, and Phe226^MACV GP1^ at the rim of the pocket (Fig. 5A). Interestingly, a tyrosine residue (Tyr106^CDR H3^) in NAb CR1-07 points toward the RBS in a similar direction as Tyr97^CDR H3^ of NAb MAC1 and forms contacts with Val117^MACV GP1^ (29) (Fig. 5B), indicative that this region of MACV GP1 is an important target for both singly reactive and cross-reactive NAbs. Indeed, we note that Val117^MACV GP1^ is also conserved in JUNV GP1 (Val117^JUNV GP1^).

The absence of a receptor-mimicking, tyrosine-mediated mode of MACV GP1 engagement by the anti-MACV NAbs MAC1 and CR1-07 may be, in part, due to the presence of a disulfide-linked insert (loop 10) unique to MACV GP1, which structurally hinders the accessibility of MAC1 and CR1-07 to the Tyr211^hTfR1^ pocket at the RBS (Fig. 5C). Superimposition of the crystal structure of MACV GP1 with those of JUNV GP1s in NAb-bound states reveals steric clashes between the MACV GP1 loop 10 and Fab CDRs of JUNV-specific NAbs (Fig. S5), supportive of the lack of MACV/JUNV cross-reactivity by NAbs GD01, OD01, and JUN1. While anti-JUNV NAb CR1-28 cross-reacts weakly with MACV GP1, it neutralizes MACV poorly (29). Similarly, a previous study has shown enhanced neutralization of MACV by mouse anti-JUNV GP antisera when MACV loop 10 was removed (36).

10.1128/mBio.02650-21.5 FIG S5 Structure overlay analysis of MACV GP1 with Fabs JUN1, OD01, GD01, and CR1-28. The MACV GP1-Fab MAC1 structure was superimposed with the structures of JUNV GP1 in complex with Fab JUN1 (A), OD01 (27) (B), GD01 (28) (C), and CR1-28 (29) (D) by aligning the GP1 subunits. MACV GP1 is shown in cartoon representation and colored yellow. Loop 10^MACV GP1^ is annotated and shown as a partially transparent surface. Fab fragments of NAbs JUN1, OD01, GD01, and CR1-28 are shown in ribbon representation, with heavy chain colored gray and light chain colored white. JUNV GP1 and Fab MAC1 are not shown. A closeup view of the steric clashes between loop 10^MACV GP1^ and Fab CDR is presented to the right of each MACV GP1-Fab model. Download FIG S5, TIF file, 2.6 MB. Copyright © 2022 Ng et al. 2022 Ng et al. https://creativecommons.org/licenses/by/4.0/ This content is distributed under the terms of the Creative Commons Attribution 4.0 International license.

The Fab MAC1 epitope is further stabilized through hydrogen bonding of paratope residues Thr99^CDR H3^ and Arg100^CDR H3^ with Val117^MACV GP1^, Lys169^MACV GP1^, and Glu171^MACV GP1^ (Fig. 5A; Fig. S3). Trp33^CDR H1^ of Fab MAC1 also contacts residue Lys170^MACV GP1^, with Asp54^CDR H2^ and Asp56^CDR H2^ forming salt bridges with Lys170^MACV GP1^ (Fig. S3). In contrast to these extensive heavy chain interactions, the Fab MAC1 light chain has a limited role in antigen recognition, with Tyr32^CDR L1^, His49^CDR L2^, Tyr50^CDR L2^, and Arg53^CDR L2^ forming minor contacts with loop 3 residues Glu121^MACV GP1^ and Tyr122^MACV GP1^ (Fig. S3).

10.1128/mBio.02650-21.3 FIG S3 Schematic diagram of contacts formed in the MACV GP1-Fab MAC1 interface. (Top) GP1-MAC1 heavy chain CDR interactions. (Bottom) GP1-MAC1 light chain CDR interactions. MACV GP1 residues are colored gray, and Fab MAC1 residues are colored green. Atoms corresponding to carbon, nitrogen, and oxygen are shown as black, blue, and red balls, respectively. Residues involved in hydrogen bonding and salt bridges are shown as sticks; residues involved in hydrophobic interactions are shown as spoked arcs. Hydrogen bonds/salt bridges and hydrophobic interactions are shown as cyan and green dotted lines, respectively. Plots were generated with LigPlot+ (69). Download FIG S3, TIF file, 2.3 MB. Copyright © 2022 Ng et al. 2022 Ng et al. https://creativecommons.org/licenses/by/4.0/ This content is distributed under the terms of the Creative Commons Attribution 4.0 International license.

Like JUNV GP1, our MACV GP1 construct encodes four N-linked glycosylation sequons, which surround the MACV GP1 RBS (Fig. 4A and B): Asn95, Asn137, Asn166, and Asn178. Similar to previous structural studies of MACV GP1 alone and in complex with hTfR1 (13, 23), electron density corresponding to at least a single GlcNAc moiety was observed at each of these sites (Fig. 4B; Fig. S4B). Interestingly, the N-linked site extending from Asn178 constitutes an ordered chain of nine glycan moieties (Man_7_GlcNAc_2_), suggestive that the di-*N*-acetylchitobiose core is protected by the surrounding proteinaceous environment during endoF1 digestion. In contrast to the glycan-independent mode of interaction observed in the JUNV GP1-Fab JUN1 complex, the terminal mannose residues of the glycan chain extending from Asn178^MACV GP1^ contribute a footprint of ∼120 Å^2^ through interaction with residues at the C-terminal segment of the Fab MAC1 CDR L2, including Leu54^MAC1^, Arg55^MAC1^, Ser56^MAC1^, Gly57^MAC1^, Val58^MAC1^, Pro59^MAC1^, and Ser60^MAC1^ (Fig. S4B). While the exact glycan composition and occupancy at Asn178^MACV GP1^ are currently unknown and warrant further investigation, the observed epitope is reminiscent of those observed on other highly glycosylated viruses such as HIV-1 Env (e.g., NAbs PGT128 and PGT135) (37) and is consistent with the requirement for NAbs to penetrate the relatively dense glycan shield presented on the arenavirus surface (31, 38).

### Anti-MACV GP1 NAbs MAC1 and CR1-07 target distinct yet overlapping epitopes.

Our MACV GP1-Fab MAC1 structure complements the MACV/JUNV cross-reactive Fab CR1-07 as the only other reported MACV GP1-bound NAb structure. To illuminate the molecular determinants that dictate NAb cross-reactivity at the NW GP1 RBS, we compared the modes of antigen recognition utilized by these two distinct NAbs (Fig. 5). While Fab MAC1 relies predominantly on the heavy chain for GP1 recognition and Fab CR1-07 relies on both heavy and light chains, both MAC1 and CR1-07 target an overlapping binding site at the rim of the central TfR1-binding pocket that includes MACV GP1 loops 3, 7, and 10 and hence preclude TfR1 recognition. Furthermore, both Fab MAC1 and Fab CR1-07 engage MACV GP1 with angles of approach that enable the NAbs to avoid clashes with loop 10, which is unique to MACV GP1 (Fig. 4 and 5).

**FIG 6:**
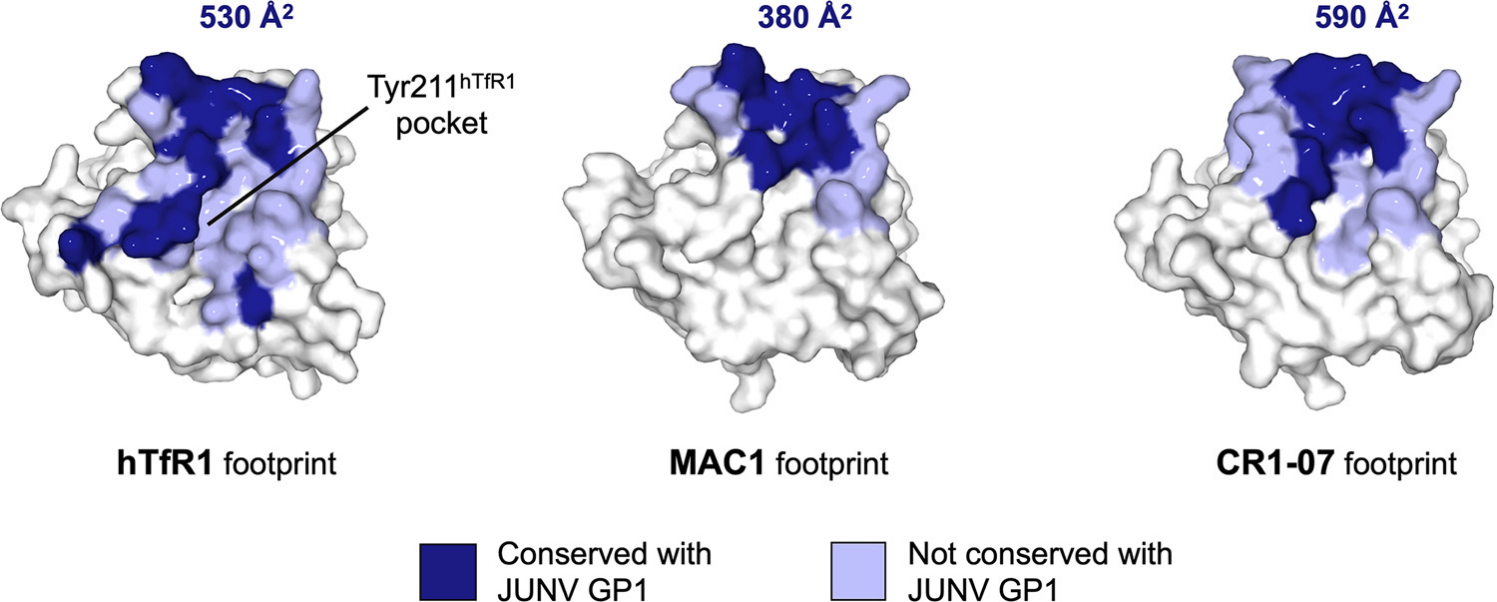
Footprints of hTfR1, Fab MAC1, and Fab CR1-07 on MACV GP1. MACV GP1 is shown in surface representation and colored white. The footprints of hTfR1 (PDB ID: 3KAS) (23), MAC1, and CR1-07 (PDB ID: 5W1M) (29) on MACV GP1 are shown in blue: dark blue represents amino acid residues conserved (identical) between MACV GP1 and JUNV GP1; nonconserved residues are colored light blue. The area of the footprints exhibited by hTfR1, MAC1, and CR1-07 on the solvent-accessible surface area of MACV GP1 by residues that are conserved with JUNV GP1 was calculated with the PDBePISA server (66), measured in buried surface area (Å^2^), and labeled accordingly.

The modes of interaction, however, are distinct and differentiated by their respective paratopes, where Fab CR1-07 contacts a significantly larger surface of MACV GP1 that is conserved between MACV and JUNV (Fig. 6). Indeed, although sequence and structural variation between MACV and JUNV GP1 at the RBS exists, ∼590 Å^2^ of the total Fab CR1-07 footprint is conserved between MACV and JUNV, compared to ∼380 Å^2^ for Fab MAC1 (Fig. 6). Thus, while structure overlay analysis does not reveal any apparent steric hindrance between Fab MAC1 and JUNV GP1, the relatively small amount of surface area that is conserved between MACV and JUNV, at the Fab MAC1 epitope, likely contributes to the absence of detectable cross-reactivity.

## DISCUSSION

JUNV and MACV, the etiological agents of Argentine and Bolivian HFs (AHF and BHF), respectively, put more than five million people at risk (39). Successful treatment of AHF by passive immunization has proven that the generation of a NAb response is crucial for controlling JUNV infection (6, 7). Characterization of the epitopes targeted by NAbs is therefore essential for the design of prophylactics and immunogens that protect against NW arenavirus infection and empower reverse vaccinology approaches (40) focused on the identification of the minimal subcomponents of the GP spike necessary for eliciting a protective immune response.

Here, we isolated eight NAbs termed JUN1 to -7 and MAC1 (Fig. 1). In our neutralization study, we used both pseudotyped lentivectors for initial MAb neutralization screening and a recombinant LCMV-based system to confirm and cross-validate our neutralization results. Interestingly, MAC1 neutralizes pseudotyped lentiviral particles with 5-fold-greater potency than that observed for the chimeric LCMV-based system. This matches previous observations on studies of Lassa virus (LASV) GP neutralization, which observed an approximately 10-fold difference between the two systems (41). Interestingly, Robinson et al. revealed that their recombinant LCMV assay yielded results very comparable to replicating wild-type LASV tested in BSL-4 (41). Therefore, while assessment of the NAbs produced here would benefit from assessment against wild-type viruses in a range of cell lines under BSL-4 containment in future studies, we envisage that our recombinant LCMV assay provides a fair surrogate for the wild-type virus neutralization. Sequence analysis reveals that the CDRs of these NAbs are distinct from the previously reported anti-NW GP1 MAb and likely originate from independent germ lines (see Tables S1 and S2 and Fig. S1 in the supplemental material). Furthermore, our structural investigation of NAbs JUN1 and MAC1 demonstrates that both NAbs target the GP1 RBS, precluding TfR1 recognition, albeit through different modes of antigen recognition (Fig. 2 to 5).

JUN1 engages the JUNV GP1 RBS by insertion of a tyrosine residue into the central pocket of the GP1, an interaction that mimics TfR1-NW GP1 recognition (23) (Fig. 2 and 3). This mode of antigen recognition has been previously observed in the structures of other structurally characterized anti-JUNV NAbs, namely, OD01 (27), GD01 (28), and CR1-28 (29) (Fig. 3). While the aforementioned anti-JUNV NAbs are derived from different germ line origins and approaches (Table S1), the varied means by which these genetically distinct antibodies converge upon a single immunological solution are consistent with previous proposals that the JUNV GP1 RBS constitutes a key site of vulnerability on the NW arenavirus surface (27–29).

Unlike JUN1, MAC1 engages the MACV GP1 RBS without occupying the central tyrosine pocket but through the interaction with peripheral residues, including those encoded in loop 10, which are unique to MACV (Fig. 4B and Fig. 5A). Consistent with the hypothesis by Clark et al. (29), our analysis indicates that this elongated region of the molecule contributes to the antigenic distinctiveness of MACV GP1 from other NW GP1s and sterically hinders JUNV GP1-specific NAbs from interacting with MACV GP1. Indeed, the inability of anti-JUNV GP1 NAbs JUN1, OD01, and GD01 to cross-react with MACV GP1 may be rationalized by structure overlay analysis, which reveals major clashes between Fab-encoded CDRs and loop 10 of MACV GP1 (Fig. S5). The differential modes of antigen recognition utilized by anti-JUNV and anti-MACV NAbs, therefore, reflect the extensive sequence and structural variation between JUNV and MACV GP1, which may have arisen following coevolution of the viruses with their individual rodent TfR1 orthologues (42).

Interestingly, despite not exhibiting any major clashes with JUNV GP1, MAC1 does not cross-react with JUNV GP1 (Fig. 1B). Comparison of MAC1 with the MACV/JUNV cross-reactive NAb, CR1-07, reveals differences between the two overlapping epitopes that may explicate this different functionality, where the latter exhibits a more extensive footprint with a greater overall surface area that is conserved between the two NW arenaviruses (Fig. 6). This observation indicates that in addition to avoiding clashes with MACV GP1 loop 10, the ability to engage a sufficiently large and conserved surface is likely also an important determinant for MACV/JUNV cross-reactivity.

Our and other NAbs recognize recombinantly produced NW GP1 (Fig. 1 to 5) (27–29), consistent with a previous study demonstrating that recombinant NW GP1 is immunogenic and capable of eliciting NAbs (43). Further, given that recombinant NW GP1 recognizes TfR1 (23, 44), these combined observations are consistent with the hypothesis that the conformations of monomeric JUNV and MACV GP1 are likely to resemble that existing on the mature trimeric NW arenaviral GP (1). The recent elucidation of the Old World (OW) Lassa virus (LASV) GP ectodomain structure (45) (“GP2-bound”) presents an opportunity to predict how NAbs JUN1 and MAC1 recognize their respective NW GP1 in the context of the higher-order GP. Similar to previous hypotheses (29, 45), our structure overlay-derived models of trimeric JUNV-GP and MACV-GP demonstrate that the RBS is directed outward and is compatible with TfR1 recognition (Fig. S6A and B). Similarly, we find that the Fab fragments from the NAbs MAC1 and JUN1 may be concurrently accommodated into each of the three GP1 components of the trimeric GP. This modeling suggests that JUN1 and MAC1 target natively accessible surfaces presented on the mature arenaviral GP (Fig. S6C and D).

10.1128/mBio.02650-21.6 FIG S6 Models of JUNV GP1-Fab JUN1, MACV GP1-Fab MAC1, and MACV GP1-hTfR1 complexes in the context of the higher-order trimeric GP assembly. (A) The crystal structure of the LASV GP1-GP2 trimer constitutes the only known structure of a higher-order arenaviral GP assembly and was used as a template to create models of trimeric NW GPs, as previously described (29, 45). JUNV GP1-Fab JUN1 and MACV GP1-Fab MAC1 were overlaid onto the structure of LASV GP1-GP2 ectodomain (PDB ID: 5VK2) (45) by aligning the GP1 subunits. All molecules are shown in cartoon representation. JUNV GP1 is colored in orange, MACV GP1 in yellow, LASV GP1 in pink, and LASV GP2 in brown. Fab JUN1 and MAC1 are not shown. (B to D) MACV GP1-hTfR1 (PDB ID: 3KAS) (23) (B), JUNV GP1-Fab JUN1 (C), and MACV GP1-Fab MAC1 (D) were overlaid onto the structure of the trimeric LASV GP1-GP2 ectodomain by aligning the GP1 subunits as in panel A. A LASV GP1 subunit is shown to provide clarity of the MACV/LASV and JUNV/LASV GP1 superimposition. Only one LASV GP2 subunit is colored brown, with the remaining two GP2 colored light gray for better visualization. LASV GP1, MACV GP1, and JUNV GP1 are colored as in panel A; hTfR1 is colored blue. Fab MAC1 and JUN1 are shown in cartoon representation, with heavy chain colored dark blue and light chain colored light blue. Download FIG S6, TIF file, 2.7 MB. Copyright © 2022 Ng et al. 2022 Ng et al. https://creativecommons.org/licenses/by/4.0/ This content is distributed under the terms of the Creative Commons Attribution 4.0 International license.

The presence of N-linked glycosylation on the surface of viral glycoproteins has been shown to play an important role in shielding the virus from the antibody-mediated immune response arising to infection (31, 46). Although not to the same level as observed in OW arenaviruses (1, 38), NW arenaviruses are extensively glycosylated (31). While the composition and occupancy of these glycans on the mature NW GP remain to be characterized, previous studies have shown that they likely occlude much of the JUNV and MACV GP surface and encircle the GP1 RBS (13, 23, 27, 31). Consistent with N-linked glycosylation providing a limited contribution to the interaction between MACV GP1 and hTfR1 (23), however, much of the RBS for both JUNV and MACV GP1s remains largely unshielded. We suggest that the exposure of this functionally important region of the NW GP surface may, in part, rationalize why each of the six structurally characterized NAbs targets the GP1 RBS. When combined with analysis of glycan occupancy and composition, future studies based upon identifying and characterizing GP2 or higher-order GP1-GP2 quaternary epitopes, such as those identified on LASV GP (41, 45, 47), will likely aid in the identification of other sites of vulnerability. Indeed, in line with this aim, several GP2-specific MAbs have been identified (41, 48, 49).

In summary, our study reveals the molecular basis for MAb-mediated neutralization of JUNV and MACV by NAbs JUN1 and MAC1, respectively. This work provides a structural template for understanding the antigenic surface of the NW GP1 and augments efforts to develop GP-specific vaccines and synergetic combinations of NAb-based prophylactics that target the NW arenavirus surface.

## MATERIALS AND METHODS

### Recombinant arenavirus engineering and mouse immunization.

JUNV-GP-chimeric LCMV (rLCMV/JUNV-GP), MACV-GP-chimeric LCMV (rLCMV/MACV-GP), JUNV-GP-chimeric PICV (rPICV/JUNV-GP), and MACV-GP-chimeric PICV (rPICV/MACV-GP) for immunization and neutralization assay testing were generated from cDNA following established protocols (50). For the rLCMV/JUNV-GP, rPICV/JUNV-GP, rLCMV/MACV-GP, and rPICV/MACV-GP constructs, the GP of strain XJ13 from JUNV (GenBank accession no. ACO52428) and the GP from the Carvallo strain of MACV (AY129248) were used, respectively. To isolate JUNV-neutralizing MAbs, C57BL/6 mice were immunized at weeks 0, 6, and 11 with rLCMV/JUNV-GP (1 × 10^7^ PFU intravenously [i.v.]) and were given a final boost at week 20 with Candid#1 (2 × 10^5^ PFU i.v.), followed by spleen cell preparation 4 days later. For the isolation of the MACV-neutralizing antibody, MAC1, and in an effort to derive cross-reactive JUNV/MACV NAbs, mice were primed at week 0 with rLCMV/MACV-GP (2 × 10^5^ PFU i.v.) and boosted at week 5 (rPICV/JUNV-GP, 10^6^ PFU i.v.) and weeks 17 and 40 (rPICV/MACV-GP, 10^6^ PFU i.v.), and spleen cells were prepared a week after the final boost. Mouse immunization experiments were performed at the University of Basel in accordance with the Swiss law for animal protection and with permission from the Veterinäramt Basel-Stadt.

### GP1-specific B cell sorting.

Fluorescence-activated cell sorting of mouse splenocytes was performed on a BD Aria II. Splenocytes were stained with anti-CD11c-phycoerythrin (PE) (BD Pharmingen), anti-F4/80-PE (eBioscience), anti-CD4-BV605 (BioLegend), anti-CD8-BV605 (BioLegend), anti-B220-BV421 (BioLegend), anti-IgD-allophycocyanin (APC)-Cy7 (BioLegend), and anti-IgM-peridinin chlorophyll protein (PerCP)-eFluor 710 (eBioscience). Biotinylated MACV and JUNV GP1 were incubated separately with both streptavidin-Alexa Fluor 488 (Thermo Fisher Scientific) and streptavidin-Alexa Fluor 647 (Thermo Fisher Scientific). CD4/CD8^−^ B220^+^ IgM^−^ IgD^−^ GP1^+^ cells were sorted into individual wells containing RNase Out (Invitrogen) and First Strand SuperScript III buffer, dithiothreitol (DTT), and H_2_O (Invitrogen), and RNA was converted into cDNA (SuperScript III reverse transcriptase; Invitrogen) using random hexamers following the manufacturer’s protocol.

### Full-length Ab cloning and expression.

The mouse antibody (Ab) variable regions of heavy and light chains were amplified with PCR using previously described primers and conditions (51). PCR products were purified and cloned into an expression vector encoding the mouse heavy or light chain constant regions using ligation-independent cloning (51). Ab variable regions were sequenced by Sanger sequencing, and germ lines were determined using IMGT, the international ImMunoGeneTics information system (http://www.imgt.org) (52).

Ab heavy and light chain plasmids were cotransfected at a 1:1 ratio into human embryonic kidney (HEK) 293F cells (Thermo Fisher Scientific) using PEI Max 40K (linear polyethylenimine hydrochloride; Polysciences, Inc.). Ab supernatants were harvested 5 days following transfection and purified using protein G affinity chromatography following the manufacturer’s protocol (GE Healthcare).

### ELISA.

ELISAs were carried out as previously described (27). High-binding ELISA 96-half-well microplates (Corning) were coated with purified JUNV GP1 or MACV GP1 (25 μL, 3 μg/mL in phosphate-buffered saline [PBS]) overnight at 4°C. Plates were washed five times with PBS containing 0.05% Tween 20 (PBS-T) and blocked with blocking buffer (5% nonfat milk in PBS-T) for 1 h at room temperature. The blocking buffer was removed, and serially diluted Ab (starting at 10 μg/mL, 1:5 dilution in blocking buffer) was added for 2 h at room temperature. Plates were washed five times with PBS-T. Secondary Ab (goat anti-mouse IgG Fc, biotin conjugate; Thermo Fisher Scientific; 1:1,000) was added for 30 min, plates were washed as described above, and alkaline phosphatase (AP)-conjugated streptavidin (1:10,000; Invitrogen) was added for 30 min. Following a final wash, *p*-nitrophenyl phosphate substrate (Sigma) was added to detect binding, and the optical densities (ODs) were measured at 405 nm.

### Competition ELISA.

High-binding ELISA 96-half-well microplates (Corning) were coated with purified JUNV GP1 (25 μL, 3 μg/mL in PBS) overnight at 4°C. Plates were washed five times with PBS-T (0.05% Tween 20) and blocked with blocking buffer (5% nonfat milk in PBS-T) for 1 h at room temperature. The blocking buffer was removed, and serially diluted JUN1 Fab (at 50 μg/mL, 1:5 dilution in blocking buffer) was added for 30 min at room temperature. JUN1 to JUN7 IgG was added at the 80% effective concentration (EC_80_) and incubated for a further 1.5 h. Plates were washed five times with PBS-T. Secondary antibody (goat anti-mouse IgG Fc biotin conjugate; Thermo Fisher Scientific; 1:1,000) was added for 30 min, plates were washed as described above, and streptavidin-AP (1:10,000; Invitrogen) was added for 30 min. Following a final wash, *p*-nitrophenyl phosphate substrate (Sigma) was added to detect binding and the ODs were measured at 405 nm. The competition is reported for JUN1 Fab at 50 μg/mL and is reported relative to the percent competition measured for JUN1 IgG.

### Pseudotyped virus preparation.

HIV-1-based virus particles, pseudotyped with JUNV GP and MACV GP, were produced in a 10-cm dish seeded the day prior with 3.5 × 10^6^ HEK293T/17 cells in 10 mL of complete Dulbecco’s modified Eagle’s medium (DMEM-C) containing 10% (vol/vol) fetal bovine serum, 100 IU/mL penicillin, and 100 μg/mL streptomycin. Cells were transfected using 45 μg of PEI (1 mg/mL; Polysciences) with 750 ng of HIV luciferase-encoding plasmid, 500 ng of HIV 8.91 gag/pol-encoding plasmid (53), and 250 ng of GP protein-encoding pHLsec vector (54). Supernatant was harvested 72 h posttransfection, filtered through a 0.45-μm filter, and stored at −80°C until required.

### Pseudovirus neutralization assays.

Serial dilutions of MAbs were prepared with DMEM and incubated with MACV or JUNV GP pseudotyped HIV-1 virus particles for 1 h at 37°C in 96-well plates. Next, HEK293T cells were added, and the plates were left for 72 h. Infection level was assessed in lysed cells with the Bright-Glo luciferase kit (Promega), using a Victor X3 multilabel reader (Perkin Elmer). IC_50_ values were calculated using GraphPad Prism.

### LCMV-based virus neutralization assays.

The neutralizing capacity of JUNV-GP and MACV-GP specific MAbs was tested in immunofocus reduction neutralization tests using chimeric engineered LCMV carrying the respective envelope glycoproteins (rLCMV/JUNV-GP; rLCMV/MACV-GP). As the cell substrate, 293T-GP cells (55) were used for optimal immunofocus formation under methylcellulose. Immunofoci were visualized using the anti-LCMV-NP MAb VL-4 (56) and were counted on an Immunospot S6 device (C.T.L.).

### GP1 and Fab expression and purification.

Constructs encoding the GP1 glycoprotein subunits of JUNV GP (residues 87 to 232; GenBank accession number ACO52428) and MACV GP (residues 87 to 257; GenBank accession number AAS77647.1) were cloned into the pHLsec mammalian expression vector (54).

HEK293T cells were transiently transfected with the desired protein constructs using PEI in the presence of the class 1 α-mannosidase inhibitor kifunensine (57). Cell supernatants were harvested 72 h after transfection and diafiltrated against a buffer containing 10 mM Tris (pH 8.0) and 150 mM NaCl (Äkta Flux diafiltration system; GE Healthcare). Glycoproteins were purified by immobilized nickel-affinity chromatography followed by size exclusion chromatography (SEC) using a Superdex 200 10/300 Increase column (GE Healthcare), equilibrated in 10 mM Tris (pH 8.0), 150 mM NaCl buffer.

The JUN1 and MAC1 Fab fragment heavy and light chain genes were PCR amplified from cDNA and cloned into the pHLsec mammalian expression vector (54). A C-terminal His_6_ tag was included in the heavy chain construct, and the two chains were coexpressed (1:1 [wt/wt] ratio of Fab heavy to light chain-expressing plasmids) in HEK293T cells and purified, as previously described (58). Fab JUN1 and MAC1 were subsequently mixed with purified JUNV GP1 and MACV GP1, respectively, in a 1:1.1 molar ratio. To aid crystallogenesis, JUNV GP1 and MACV GP1 were partially deglycosylated with endoF1 (25°C, 18 h). Following deglycosylation, the GP1-Fab complexes were repurified by SEC, as described above.

### Data collection and structure determination.

Crystallization experiments were performed at room temperature using the sitting drop vapor diffusion method (59). Crystals of JUNV GP1-Fab JUN1 complex were obtained by mixing 100 nL of a 5.9-mg/mL protein sample and 100 nL of precipitant containing 20% (wt/vol) polyethylene glycol (PEG) 8000 and 0.1 M HEPES, pH 7.5. Crystals of MACV GP1-Fab MAC1 complex were obtained by mixing 100 nL of an 8.8-mg/mL protein sample and 100 nL of precipitant containing 1.4 M sodium malonate, pH 6.0. Crystallization drops were equilibrated against 95 μL of a precipitant-containing reservoir. In all instances, crystals were cryoprotected by transfer into a solution of the respective precipitant supplemented with 25% (vol/vol) glycerol, prior to flash cooling in liquid nitrogen.

X-ray diffraction data were recorded at Diamond Light Source, United Kingdom. Crystal data were indexed, integrated, and scaled with XIA2 (60). The structure of JUNV GP1-Fab JUN1 complex was solved by molecular replacement with PHASER (61), using the crystal structures of JUNV GP1 and Fab OD01 (PDB: 5NUZ [27]) as search models. The structure of MACV GP1-Fab MAC1 complex was phased by molecular replacement with PHASER (61) using MACV GP1 (PDB: 2WFO [13]) and Fab OD01 (PDB: 5NUZ [27]) as search models. For all structures, iterative rounds of model building and refinement were performed using COOT (62) and PHENIX with TLS parameterization (63), respectively. MolProbity (64) was used to validate model quality. Data collection and refinement statistics are presented in Table S3 in the supplemental material. The PyMOL molecular graphics system (https://www.schrodinger.com/pymol) was used to generate the structural models presented in the figures.

### Statistical analysis.

The assays presented in Fig. 1B to E were performed in duplicate and repeated at least twice. All data points presented in Fig. 1B to D are expressed as means. All data points are shown in Fig. 1E. Error bars represent the range of the values for experiments performed in duplicate.

### Data and material availability.

All data needed to evaluate the conclusions in the paper are present in the paper and/or the supplemental material. Coordinates and structure factors of MACV GP1-Fab MAC1 and JUNV GP1-Fab JUN1 have been deposited in the Protein Data Bank with the accession codes 7QU1 and 7QU2, respectively.

## References

[1] Pryce R, Ng WM, Zeltina A, Watanabe Y, Omari KE, Wagner A, Bowden TA. 2019. Structure-based classification defines the discrete conformational classes adopted by the arenaviral GP1. J Virol 93:e01048-18. doi:10.1128/JVI.01048-18. PMC6288339 30305351

[2] Paessler S, Walker DH. 2013. Pathogenesis of the viral hemorrhagic fevers. Annu Rev Pathol 8:411–440. doi:10.1146/annurev-pathol-020712-164041. 23121052

[3] Maiztegui JI, McKee KT, Jr, Barrera Oro JG, Harrison LH, Gibbs PH, Feuillade MR, Enria DA, Briggiler AM, Levis SC, Ambrosio AM, Halsey NA, Peters CJ. 1998. Protective efficacy of a live attenuated vaccine against Argentine hemorrhagic fever. AHF Study Group. J Infect Dis 177:277–283. doi:10.1086/514211. 9466512

[4] Enria DA, Briggiler AM, Sánchez Z. 2008. Treatment of Argentine hemorrhagic fever. Antiviral Res 78:132–139. doi:10.1016/j.antiviral.2007.10.010. 18054395 PMC7144853

[5] Johnson DM, Jokinen JD, Wang M, Pfeffer T, Tretyakova I, Carrion R, Jr, Griffiths A, Pushko P, Lukashevich IS. 2020. Bivalent Junin & Machupo experimental vaccine based on alphavirus RNA replicon vector. Vaccine 38:2949–2959. doi:10.1016/j.vaccine.2020.02.053. 32111526 PMC7112472

[6] Maiztegui JI, Fernandez NJ, de Damilano AJ. 1979. Efficacy of immune plasma in treatment of Argentine haemorrhagic fever and association between treatment and a late neurological syndrome. Lancet ii:1216–1217. doi:10.1016/S0140-6736(79)92335-3. 92624

[7] Enria DA, Briggiler AM, Fernandez NJ, Levis SC, Maiztegui JI. 1984. Importance of dose of neutralising antibodies in treatment of Argentine haemorrhagic fever with immune plasma. Lancet ii:255–256. doi:10.1016/S0140-6736(84)90299-X. 6146809

[8] Crispin M, Zeltina A, Zitzmann N, Bowden TA. 2016. Native functionality and therapeutic targeting of arenaviral glycoproteins. Curr Opin Virol 18:70–75. doi:10.1016/j.coviro.2016.04.001. 27104809 PMC4983490

[9] Nunberg JH, York J. 2012. The curious case of arenavirus entry, and its inhibition. Viruses 4:83–101. doi:10.3390/v4010083. 22355453 PMC3280523

[10] York J, Nunberg JH. 2016. Myristoylation of the arenavirus envelope glycoprotein stable signal peptide is critical for membrane fusion but dispensable for virion morphogenesis. J Virol 90:8341–8350. doi:10.1128/JVI.01124-16. 27412594 PMC5008094

[11] York J, Romanowski V, Lu M, Nunberg JH. 2004. The signal peptide of the Junin arenavirus envelope glycoprotein is myristoylated and forms an essential subunit of the mature G1-G2 complex. J Virol 78:10783–10792. doi:10.1128/JVI.78.19.10783-10792.2004. 15367645 PMC516395

[12] Bowden TA, Jones EY, Stuart DI. 2011. Cells under siege: viral glycoprotein interactions at the cell surface. J Struct Biol 175:120–126. doi:10.1016/j.jsb.2011.03.016. 21440638 PMC3137789

[13] Bowden TA, Crispin M, Graham SC, Harvey DJ, Grimes JM, Jones EY, Stuart DI. 2009. Unusual molecular architecture of the Machupo virus attachment glycoprotein. J Virol 83:8259–8265. doi:10.1128/JVI.00761-09. 19494008 PMC2715760

[14] Igonet S, Vaney M-C, Vonrhein C, Vonhrein C, Bricogne G, Stura EA, Hengartner H, Eschli B, Rey FA. 2011. X-ray structure of the arenavirus glycoprotein GP2 in its postfusion hairpin conformation. Proc Natl Acad Sci USA 108:19967–19972. doi:10.1073/pnas.1108910108. 22123988 PMC3250147

[15] Parsy ML, Harlos K, Huiskonen JT, Bowden TA. 2013. Crystal structure of Venezuelan hemorrhagic fever virus fusion glycoprotein reveals a class 1 postfusion architecture with extensive glycosylation. J Virol 87:13070–13075. doi:10.1128/JVI.02298-13. 24049182 PMC3838125

[16] Hulswit RJG, Paesen GC, Bowden TA, Shi X. 2021. Recent advances in bunyavirus glycoprotein research: precursor processing, receptor binding and structure. Viruses 13:353. doi:10.3390/v13020353. 33672327 PMC7926653

[17] Goncalves AR, Moraz ML, Pasquato A, Helenius A, Lozach PY, Kunz S. 2013. Role of DC-SIGN in Lassa virus entry into human dendritic cells. J Virol 87:11504–11515. doi:10.1128/JVI.01893-13. 23966408 PMC3807329

[18] Shimojima M, Stroher U, Ebihara H, Feldmann H, Kawaoka Y. 2012. Identification of cell surface molecules involved in dystroglycan-independent Lassa virus cell entry. J Virol 86:2067–2078. doi:10.1128/JVI.06451-11. 22156524 PMC3302412

[19] Flanagan ML, Oldenburg J, Reignier T, Holt N, Hamilton GA, Martin VK, Cannon PM. 2008. New world clade B arenaviruses can use transferrin receptor 1 (TfR1)-dependent and -independent entry pathways, and glycoproteins from human pathogenic strains are associated with the use of TfR1. J Virol 82:938–948. doi:10.1128/JVI.01397-07. 18003730 PMC2224602

[20] Sarute N, Ross SR. 2017. New World arenavirus biology. Annu Rev Virol 4:141–158. doi:10.1146/annurev-virology-101416-042001. 28645238 PMC7478856

[21] Abraham J, Kwong JA, Albarino CG, Lu JG, Radoshitzky SR, Salazar-Bravo J, Farzan M, Spiropoulou CF, Choe H. 2009. Host-species transferrin receptor 1 orthologs are cellular receptors for nonpathogenic new world clade B arenaviruses. PLoS Pathog 5:e1000358. doi:10.1371/journal.ppat.1000358. 19343214 PMC2658809

[22] Radoshitzky SR, Abraham J, Spiropoulou CF, Kuhn JH, Nguyen D, Li W, Nagel J, Schmidt PJ, Nunberg JH, Andrews NC, Farzan M, Choe H. 2007. Transferrin receptor 1 is a cellular receptor for New World haemorrhagic fever arenaviruses. Nature 446:92–96. doi:10.1038/nature05539. 17287727 PMC3197705

[23] Abraham J, Corbett KD, Farzan M, Choe H, Harrison SC. 2010. Structural basis for receptor recognition by New World hemorrhagic fever arenaviruses. Nat Struct Mol Biol 17:438–444. doi:10.1038/nsmb.1772. 20208545 PMC2920743

[24] Li S, Sun Z, Pryce R, Parsy ML, Fehling SK, Schlie K, Siebert CA, Garten W, Bowden TA, Strecker T, Huiskonen JT. 2016. Acidic pH-induced conformations and LAMP1 binding of the Lassa virus glycoprotein spike. PLoS Pathog 12:e1005418. doi:10.1371/journal.ppat.1005418. 26849049 PMC4743923

[25] Branco LM, Grove JN, Moses LM, Goba A, Fullah M, Momoh M, Schoepp RJ, Bausch DG, Garry RF. 2010. Shedding of soluble glycoprotein 1 detected during acute Lassa virus infection in human subjects. Virol J 7:306. doi:10.1186/1743-422X-7-306. 21062490 PMC2993672

[26] Lawrence CM, Ray S, Babyonyshev M, Galluser R, Borhani DW, Harrison SC. 1999. Crystal structure of the ectodomain of human transferrin receptor. Science 286:779–782. doi:10.1126/science.286.5440.779. 10531064

[27] Zeltina A, Krumm SA, Sahin M, Struwe WB, Harlos K, Nunberg JH, Crispin M, Pinschewer DD, Doores KJ, Bowden TA. 2017. Convergent immunological solutions to Argentine hemorrhagic fever virus neutralization. Proc Natl Acad Sci USA 114:7031–7036. doi:10.1073/pnas.1702127114. 28630325 PMC5502616

[28] Mahmutovic S, Clark L, Levis SC, Briggiler AM, Enria DA, Harrison SC, Abraham J. 2015. Molecular basis for antibody-mediated neutralization of New World hemorrhagic fever mammarenaviruses. Cell Host Microbe 18:705–713. doi:10.1016/j.chom.2015.11.005. 26651946 PMC4685251

[29] Clark LE, Mahmutovic S, Raymond DD, Dilanyan T, Koma T, Manning JT, Shankar S, Levis SC, Briggiler AM, Enria DA, Wucherpfennig KW, Paessler S, Abraham J. 2018. Vaccine-elicited receptor-binding site antibodies neutralize two New World hemorrhagic fever arenaviruses. Nat Commun 9:1884. doi:10.1038/s41467-018-04271-z. 29760382 PMC5951886

[30] Zeitlin L, Cross RW, Geisbert JB, Borisevich V, Agans KN, Prasad AN, Enterlein S, Aman MJ, Bornholdt ZA, Brennan MB, Campbell L, Kim D, Mlakar N, Moyer CL, Pauly MH, Shestowsky W, Whaley KJ, Fenton KA, Geisbert TW. 2021. Therapy for Argentine hemorrhagic fever in nonhuman primates with a humanized monoclonal antibody. Proc Natl Acad Sci USA 118:e2023332118. doi:10.1073/pnas.2023332118. 33836604 PMC7980402

[31] Sommerstein R, Flatz L, Remy MM, Malinge P, Magistrelli G, Fischer N, Sahin M, Bergthaler A, Igonet S, Ter Meulen J, Rigo D, Meda P, Rabah N, Coutard B, Bowden TA, Lambert PH, Siegrist CA, Pinschewer DD. 2015. Arenavirus glycan shield promotes neutralizing antibody evasion and protracted infection. PLoS Pathog 11:e1005276. doi:10.1371/journal.ppat.1005276. 26587982 PMC4654586

[32] Bergthaler A, Gerber NU, Merkler D, Horvath E, de la Torre JC, Pinschewer DD. 2006. Envelope exchange for the generation of live-attenuated arenavirus vaccines. PLoS Pathog 2:e51. doi:10.1371/journal.ppat.0020051. 16751848 PMC1472708

[33] Sanchez A, Pifat DY, Kenyon RH, Peters CJ, McCormick JB, Kiley MP. 1989. Junin virus monoclonal antibodies: characterization and cross-reactivity with other arenaviruses. J Gen Virol 70:1125–1132. doi:10.1099/0022-1317-70-5-1125. 2471803

[34] Zeitlin L, Geisbert JB, Deer DJ, Fenton KA, Bohorov O, Bohorova N, Goodman C, Kim D, Hiatt A, Pauly MH, Velasco J, Whaley KJ, Altmann F, Gruber C, Steinkellner H, Honko AN, Kuehne AI, Aman MJ, Sahandi S, Enterlein S, Zhan X, Enria D, Geisbert TW. 2016. Monoclonal antibody therapy for Junin virus infection. Proc Natl Acad Sci USA 113:4458–4463. doi:10.1073/pnas.1600996113. 27044104 PMC4843420

[35] Martin AC, Thornton JM. 1996. Structural families in loops of homologous proteins: automatic classification, modelling and application to antibodies. J Mol Biol 263:800–815. doi:10.1006/jmbi.1996.0617. 8947577

[36] Brouillette RB, Phillips EK, Ayithan N, Maury W. 2017. Differences in glycoprotein complex (GPC) receptor binding site accessibility prompt poor cross-reactivity of neutralizing antibodies between closely related arenaviruses. J Virol 91:e01454-16. doi:10.1128/JVI.01454-16. 28100617 PMC5355595

[37] Doores KJ. 2015. The HIV glycan shield as a target for broadly neutralizing antibodies. FEBS J 282:4679–4691. doi:10.1111/febs.13530. 26411545 PMC4950053

[38] Watanabe Y, Raghwani J, Allen JD, Seabright GE, Li S, Moser F, Huiskonen JT, Strecker T, Bowden TA, Crispin M. 2018. Structure of the Lassa virus glycan shield provides a model for immunological resistance. Proc Natl Acad Sci USA 115:7320–7325. doi:10.1073/pnas.1803990115. 29941589 PMC6048489

[39] Mills JN, Ellis BA, Childs JE, McKee KT, Jr, Maiztegui JI, Peters CJ, Ksiazek TG, Jahrling PB. 1994. Prevalence of infection with Junin virus in rodent populations in the epidemic area of Argentine hemorrhagic fever. Am J Trop Med Hyg 51:554–562. doi:10.4269/ajtmh.1994.51.554. 7985747

[40] Burton DR. 2017. What are the most powerful immunogen design vaccine strategies? Reverse vaccinology 2.0 shows great promise. Cold Spring Harb Perspect Biol 9:a030262. doi:10.1101/cshperspect.a030262. 28159875 PMC5540812

[41] Robinson JE, Hastie KM, Cross RW, Yenni RE, Elliott DH, Rouelle JA, Kannadka CB, Smira AA, Garry CE, Bradley BT, Yu H, Shaffer JG, Boisen ML, Hartnett JN, Zandonatti MA, Rowland MM, Heinrich ML, Martinez-Sobrido L, Cheng B, de la Torre JC, Andersen KG, Goba A, Momoh M, Fullah M, Gbakie M, Kanneh L, Koroma VJ, Fonnie R, Jalloh SC, Kargbo B, Vandi MA, Gbetuwa M, Ikponmwosa O, Asogun DA, Okokhere PO, Follarin OA, Schieffelin JS, Pitts KR, Geisbert JB, Kulakoski PC, Wilson RB, Happi CT, Sabeti PC, Gevao SM, Khan SH, Grant DS, Geisbert TW, Saphire EO, Branco LM, Garry RF. 2016. Most neutralizing human monoclonal antibodies target novel epitopes requiring both Lassa virus glycoprotein subunits. Nat Commun 7:11544. doi:10.1038/ncomms11544. 27161536 PMC4866400

[42] Choe H, Jemielity S, Abraham J, Radoshitzky SR, Farzan M. 2011. Transferrin receptor 1 in the zoonosis and pathogenesis of New World hemorrhagic fever arenaviruses. Curr Opin Microbiol 14:476–482. doi:10.1016/j.mib.2011.07.014. 21807555 PMC3159852

[43] Borenstein-Katz A, Shulman A, Hamawi H, Leitner O, Diskin R. 2019. Differential antibody-based immune response against isolated GP1 receptor-binding domains from Lassa and Junin viruses. J Virol 93:e00090-19. doi:10.1128/JVI.00090-19. 30728269 PMC6450128

[44] Cohen-Dvashi H, Amon R, Agans KN, Cross RW, Borenstein-Katz A, Mateo M, Baize S, Padler-Karavani V, Geisbert TW, Diskin R. 2020. Rational design of universal immunotherapy for TfR1-tropic arenaviruses. Nat Commun 11:67. doi:10.1038/s41467-019-13924-6. 31900422 PMC6941993

[45] Hastie KM, Zandonatti MA, Kleinfelter LM, Heinrich ML, Rowland MM, Chandran K, Branco LM, Robinson JE, Garry RF, Saphire EO. 2017. Structural basis for antibody-mediated neutralization of Lassa virus. Science 356:923–928. doi:10.1126/science.aam7260. 28572385 PMC6007842

[46] Watanabe Y, Bowden TA, Wilson IA, Crispin M. 2019. Exploitation of glycosylation in enveloped virus pathobiology. Biochim Biophys Acta Gen Subj 1863:1480–1497. doi:10.1016/j.bbagen.2019.05.012. 31121217 PMC6686077

[47] Zeltina A, Bowden TA. 2017. Human antibody pieces together the puzzle of the trimeric Lassa virus surface antigen. Nat Struct Mol Biol 24:559–560. doi:10.1038/nsmb.3431. 28686227

[48] Amanat F, Duehr J, Oestereich L, Hastie KM, Ollmann Saphire E, Krammer F. 2018. Antibodies to the glycoprotein GP2 subunit cross-react between Old and New World arenaviruses. mSphere 3:e00189-18. doi:10.1128/mSphere.00189-18. 29720525 PMC5932378

[49] Ruo SL, Mitchell SW, Kiley MP, Roumillat LF, Fisher-Hoch SP, McCormick JB. 1991. Antigenic relatedness between arenaviruses defined at the epitope level by monoclonal antibodies. J Gen Virol 72:549–555. doi:10.1099/0022-1317-72-3-549. 1706408

[50] Bonilla WV, Kirchhammer N, Marx AF, Kallert SM, Krzyzaniak MA, Lu M, Darbre S, Schmidt S, Raguz J, Berka U, Vincenti I, Pauzuolis M, Kerber R, Hoepner S, Günther S, Magnus C, Merkler D, Orlinger KK, Zippelius A, Pinschewer DD. 2021. Heterologous arenavirus vector prime-boost overrules self-tolerance for efficient tumor-specific CD8 T cell attack. Cell Rep Med 2:100209. doi:10.1016/j.xcrm.2021.100209. 33763654 PMC7974551

[51] von Boehmer L, Liu C, Ackerman S, Gitlin AD, Wang Q, Gazumyan A, Nussenzweig MC. 2016. Sequencing and cloning of antigen-specific antibodies from mouse memory B cells. Nat Protoc 11:1908–1923. doi:10.1038/nprot.2016.102. 27658009

[52] Lefranc MP, Giudicelli V, Duroux P, Jabado-Michaloud J, Folch G, Aouinti S, Carillon E, Duvergey H, Houles A, Paysan-Lafosse T, Hadi-Saljoqi S, Sasorith S, Lefranc G, Kossida S. 2015. IMGT, the international ImMunoGeneTics information system 25 years on. Nucleic Acids Res 43:D413–D422. doi:10.1093/nar/gku1056. 25378316 PMC4383898

[53] Zufferey R, Nagy D, Mandel RJ, Naldini L, Trono D. 1997. Multiply attenuated lentiviral vector achieves efficient gene delivery in vivo. Nat Biotechnol 15:871–875. doi:10.1038/nbt0997-871. 9306402

[54] Aricescu AR, Lu W, Jones EY. 2006. A time- and cost-efficient system for high-level protein production in mammalian cells. Acta Crystallogr D Biol Crystallogr 62:1243–1250. doi:10.1107/S0907444906029799. 17001101

[55] Flatz L, Hegazy AN, Bergthaler A, Verschoor A, Claus C, Fernandez M, Gattinoni L, Johnson S, Kreppel F, Kochanek S, Broek M, Radbruch A, Lévy F, Lambert PH, Siegrist CA, Restifo NP, Löhning M, Ochsenbein AF, Nabel GJ, Pinschewer DD. 2010. Development of replication-defective lymphocytic choriomeningitis virus vectors for the induction of potent CD8+ T cell immunity. Nat Med 16:339–345. doi:10.1038/nm.2104. 20139992 PMC3247638

[56] Battegay M, Cooper S, Althage A, Bänziger J, Hengartner H, Zinkernagel RM. 1991. Quantification of lymphocytic choriomeningitis virus with an immunological focus assay in 24- or 96-well plates. J Virol Methods 33:191–198. doi:10.1016/0166-0934(91)90018-u. 1939506

[57] Elbein AD, Tropea JE, Mitchell M, Kaushal GP. 1990. Kifunensine, a potent inhibitor of the glycoprotein processing mannosidase I. J Biol Chem 265:15599–15605. doi:10.1016/S0021-9258(18)55439-9. 2144287

[58] Avanzato VA, Oguntuyo KY, Escalera-Zamudio M, Gutierrez B, Golden M, Kosakovsky Pond SL, Pryce R, Walter TS, Seow J, Doores KJ, Pybus OG, Munster VJ, Lee B, Bowden TA. 2019. A structural basis for antibody-mediated neutralization of Nipah virus reveals a site of vulnerability at the fusion glycoprotein apex. Proc Natl Acad Sci USA 116:25057–25067. doi:10.1073/pnas.1912503116. 31767754 PMC6911215

[59] Walter TS, Diprose JM, Mayo CJ, Siebold C, Pickford MG, Carter L, Sutton GC, Berrow NS, Brown J, Berry IM, Stewart-Jones GB, Grimes JM, Stammers DK, Esnouf RM, Jones EY, Owens RJ, Stuart DI, Harlos K. 2005. A procedure for setting up high-throughput nanolitre crystallization experiments. Crystallization workflow for initial screening, automated storage, imaging and optimization. Acta Crystallogr D Biol Crystallogr 61:651–657. doi:10.1107/S0907444905007808. 15930615 PMC7159505

[60] Winter G. 2010. xia2: an expert system for macromolecular crystallography data reduction. J Appl Crystallogr 43:186–190. doi:10.1107/S0021889809045701.

[61] McCoy AJ, Gross-Kunstleve RW, Adams PD, Winn MD, Storoni LC, Read RJ. 2007. Phaser crystallographic software. J Appl Crystallogr 40:658–674. doi:10.1107/S0021889807021206. 19461840 PMC2483472

[62] Emsley P, Cowtan K. 2004. Coot: model-building tools for molecular graphics. Acta Crystallogr D Biol Crystallogr 60:2126–2132. doi:10.1107/S0907444904019158. 15572765

[63] Adams PD, Grosse-Kunstleve RW, Hung LW, Ioerger TR, McCoy AJ, Moriarty NW, Read RJ, Sacchettini JC, Sauter NK, Terwilliger TC. 2002. PHENIX: building new software for automated crystallographic structure determination. Acta Crystallogr D Biol Crystallogr 58:1948–1954. doi:10.1107/S0907444902016657. 12393927

[64] Chen VB, Arendall WB, III, Headd JJ, Keedy DA, Immormino RM, Kapral GJ, Murray LW, Richardson JS, Richardson DC. 2010. MolProbity: all-atom structure validation for macromolecular crystallography. Acta Crystallogr D Biol Crystallogr 66:12–21. doi:10.1107/S0907444909042073. 20057044 PMC2803126

[65] Ren J, Wen L, Gao X, Jin C, Xue Y, Yao X. 2009. DOG 1.0: illustrator of protein domain structures. Cell Res 19:271–273. doi:10.1038/cr.2009.6. 19153597

[66] Krissinel E, Henrick K. 2007. Inference of macromolecular assemblies from crystalline state. J Mol Biol 372:774–797. doi:10.1016/j.jmb.2007.05.022. 17681537

[67] Corpet F. 1988. Multiple sequence alignment with hierarchical clustering. Nucleic Acids Res 16:10881–10890. doi:10.1093/nar/16.22.10881. 2849754 PMC338945

[68] Robert X, Gouet P. 2014. Deciphering key features in protein structures with the new ENDscript server. Nucleic Acids Res 42:W320–W324. doi:10.1093/nar/gku316. 24753421 PMC4086106

[69] Laskowski RA, Swindells MB. 2011. LigPlot+: multiple ligand-protein interaction diagrams for drug discovery. J Chem Inf Model 51:2778–2786. doi:10.1021/ci200227u. 21919503

