# Supplementary figures and images for: Contrasting Modes of New World Arenavirus Neutralization by Immunization-Elicited Monoclonal Antibodies
Source: mBio. 2022 Mar 22;13(2):e02650-21. doi: 10.1128/mbio.02650-21 (PMC9040744; doi:10.1128/mbio.02650-21)

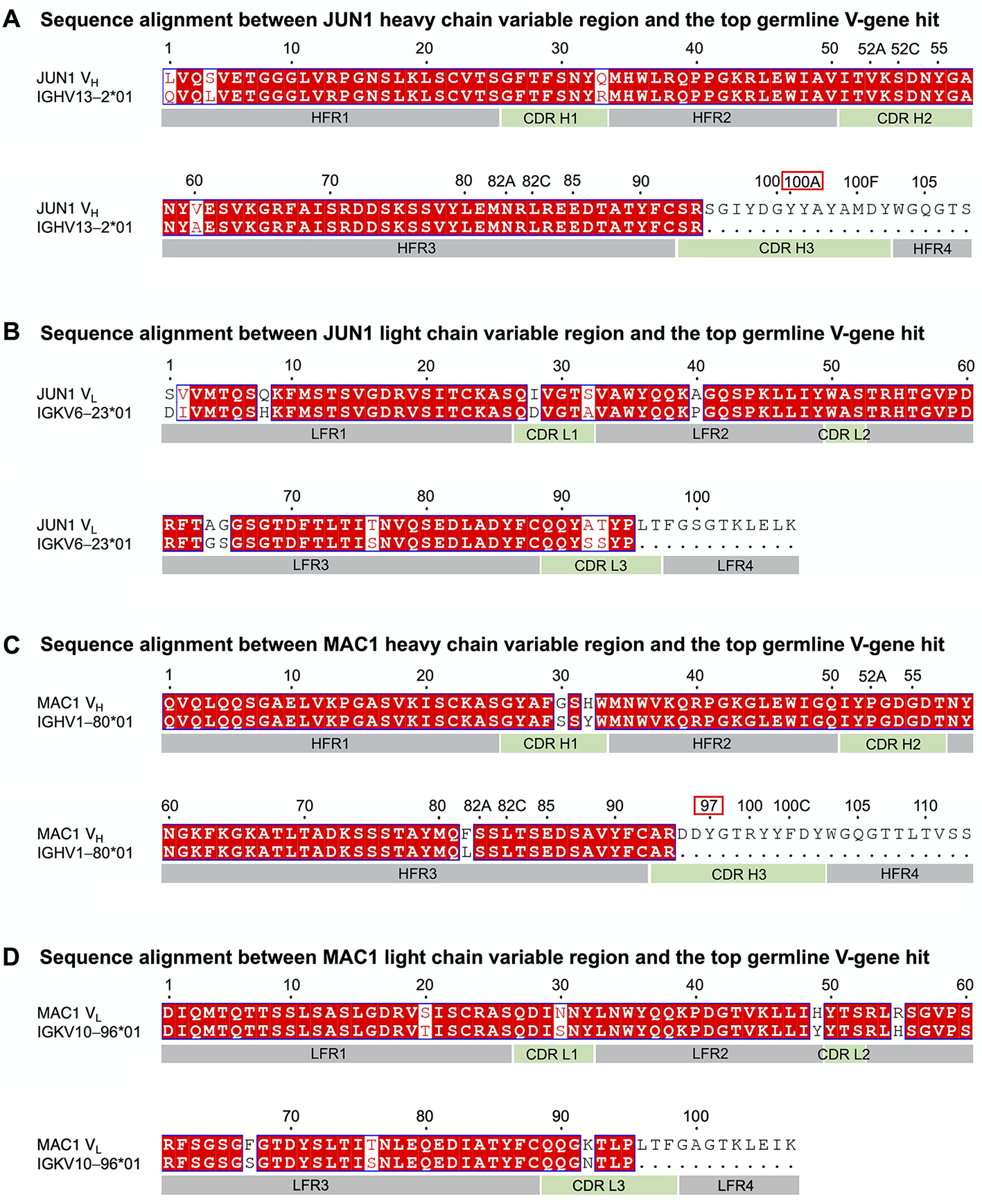

Supplement: FIG S1 [file mbio.02650-21-sf001.tif]

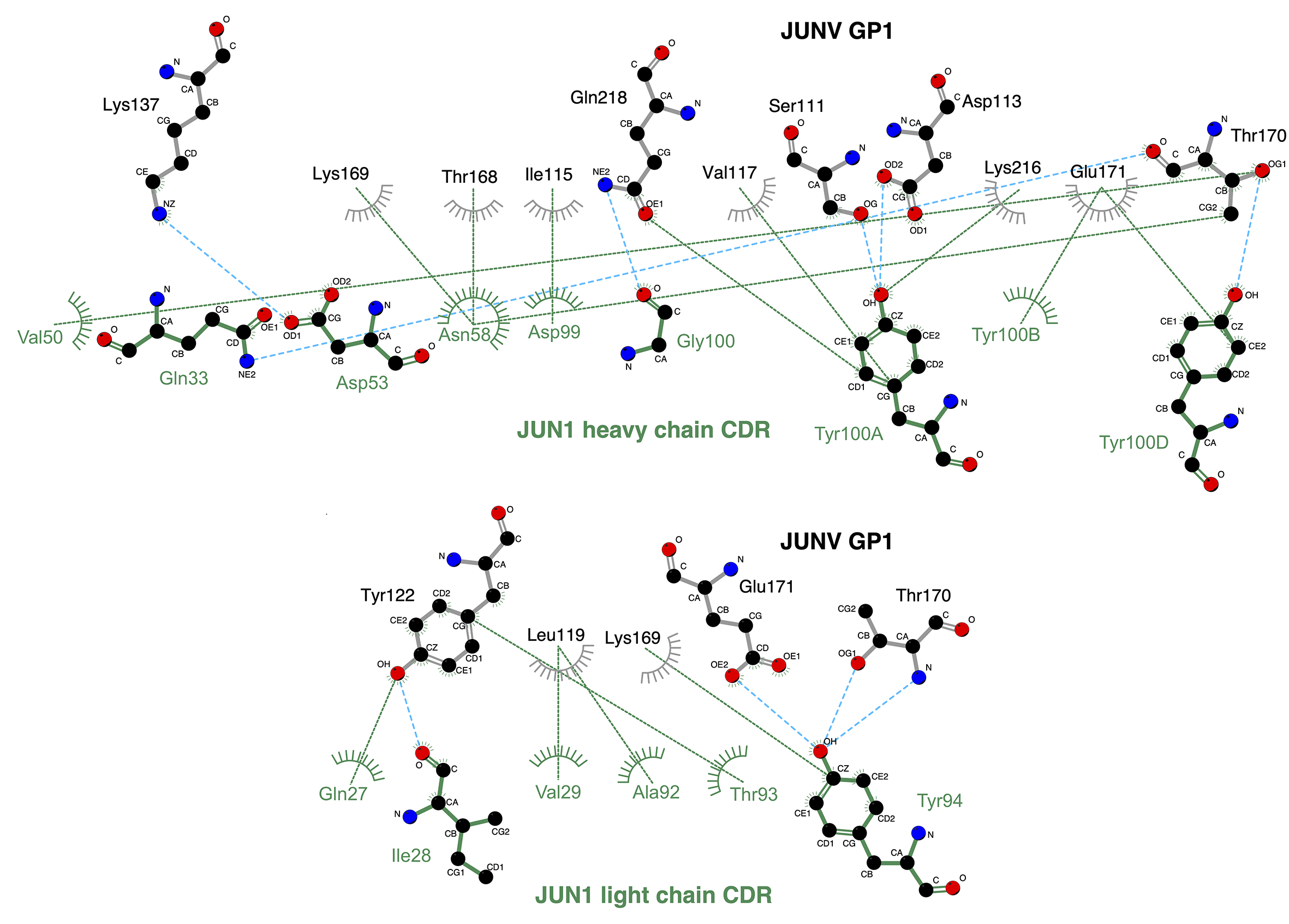

Supplement: FIG S2 [file mbio.02650-21-sf002.tif]

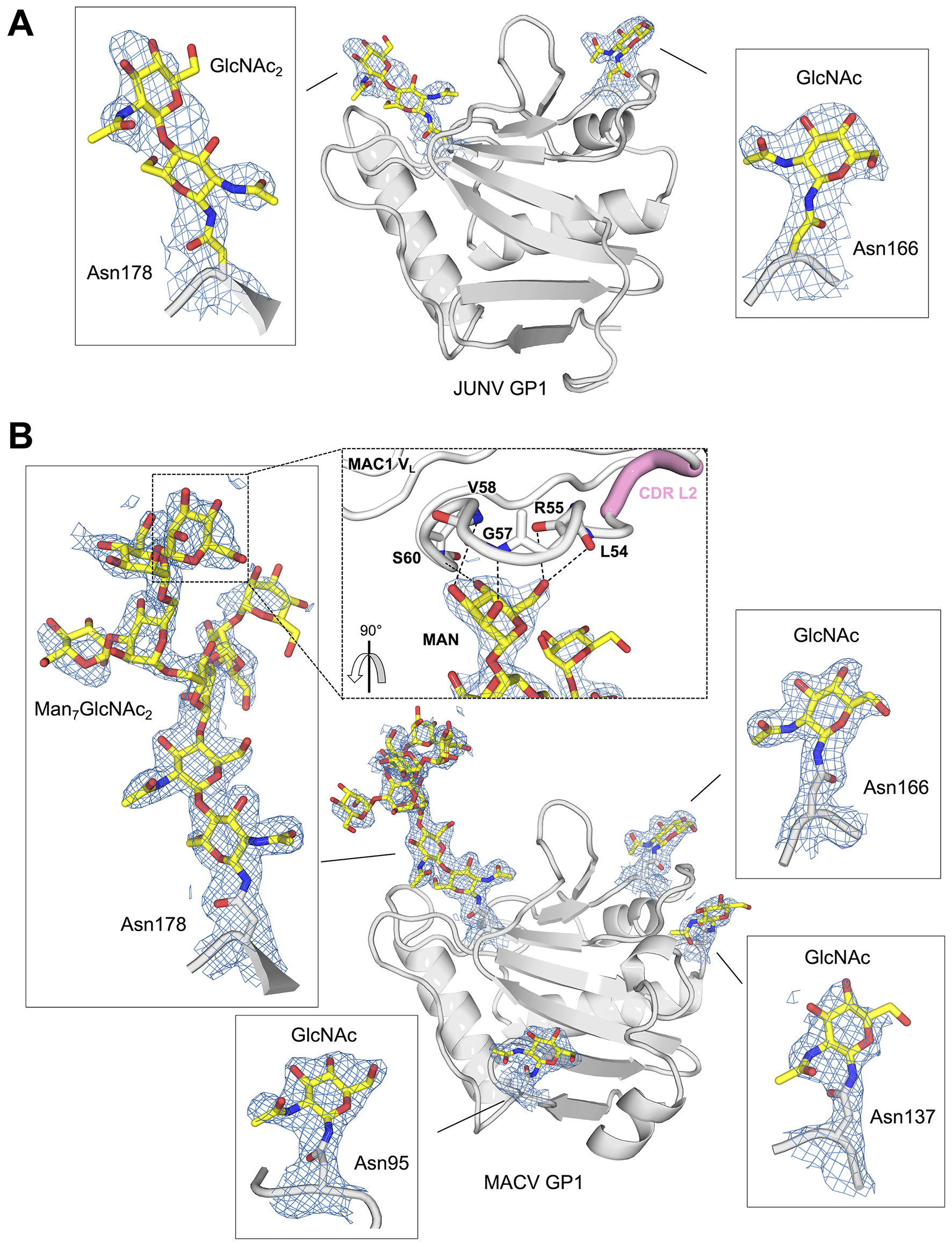

Supplement: FIG S4 [file mbio.02650-21-sf004.tif]

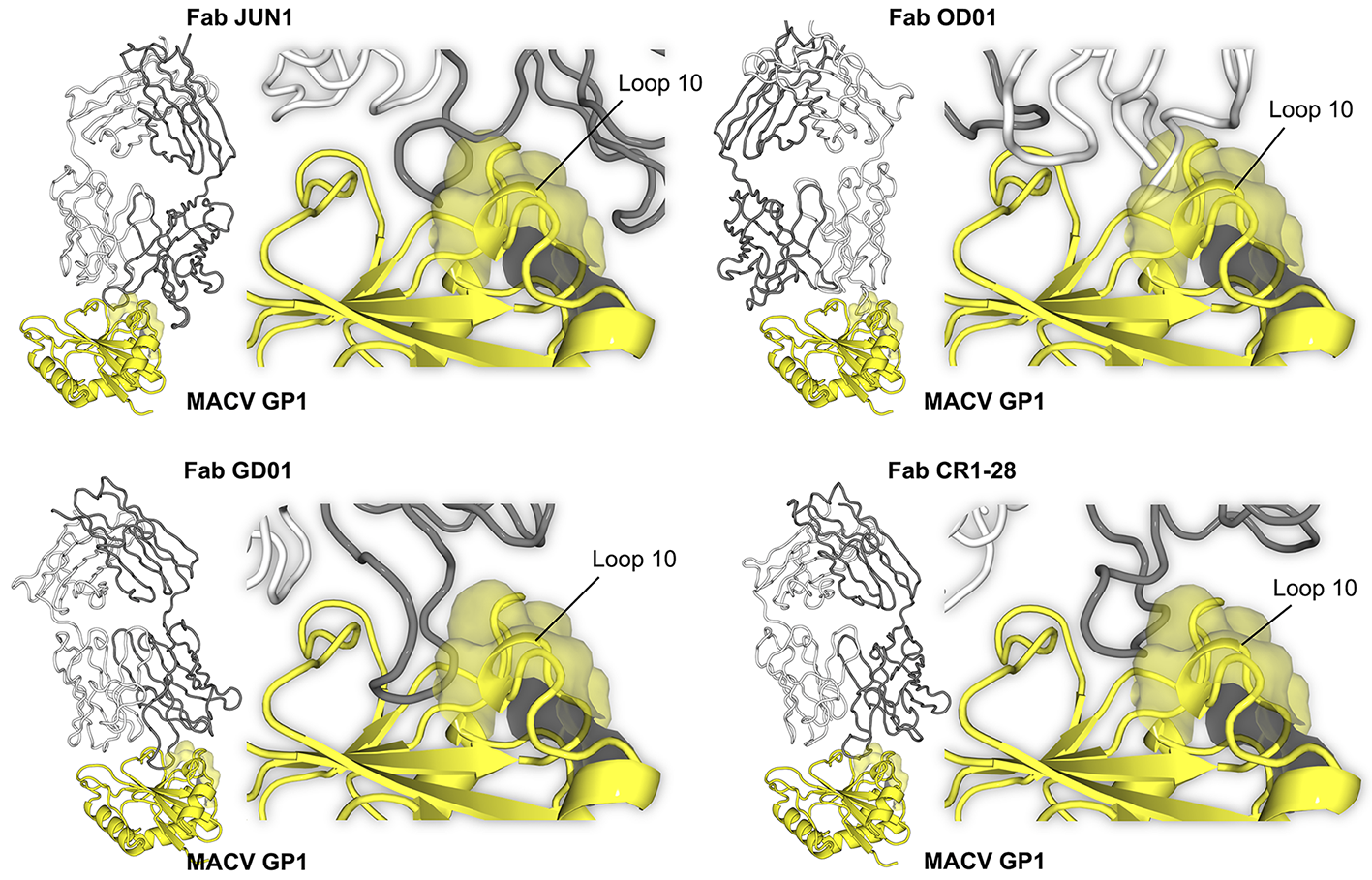

Supplement: FIG S5 [file mbio.02650-21-sf005.tif]

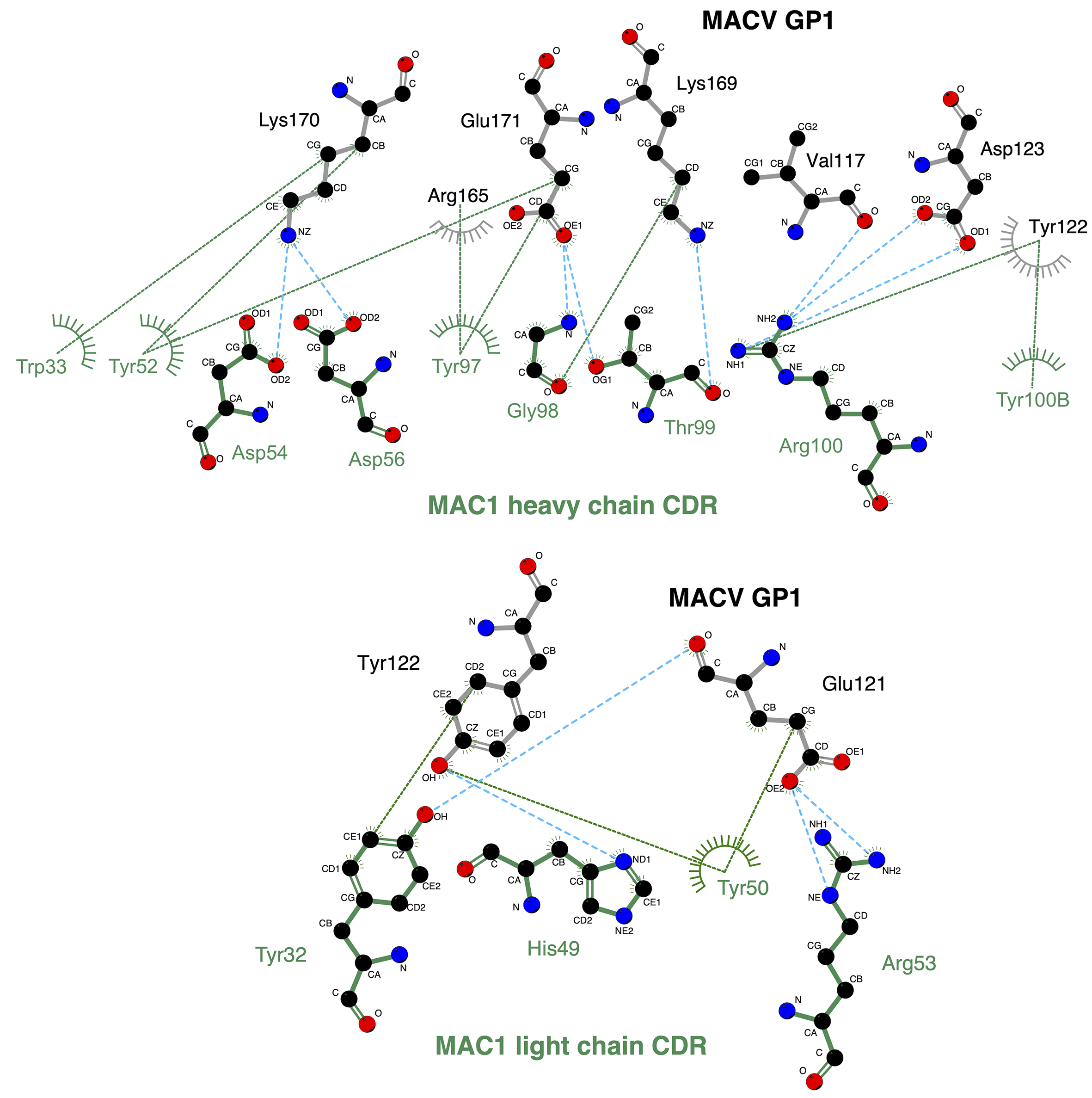

Supplement: FIG S3 [file mbio.02650-21-sf003.tif]

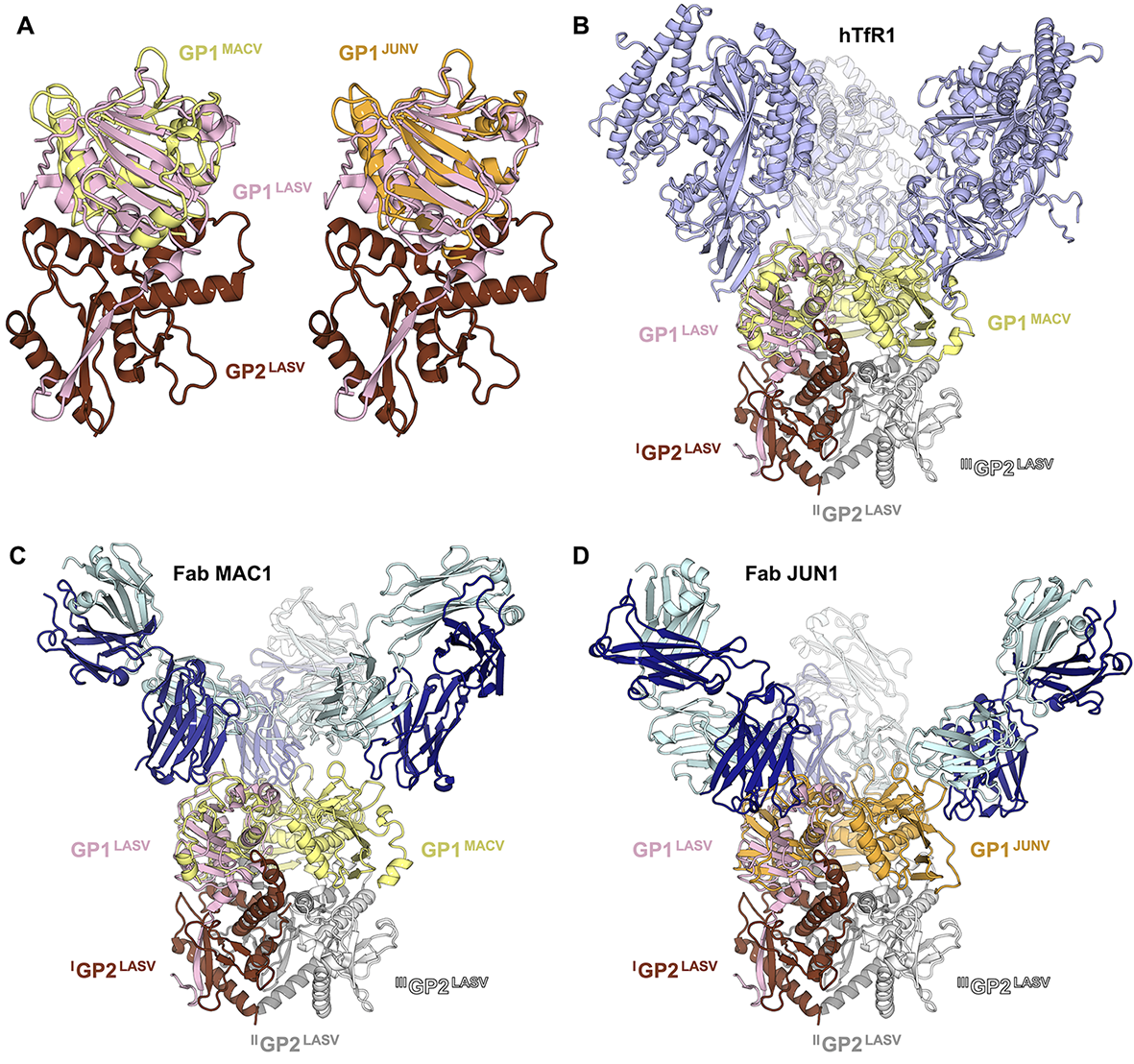

Supplement: FIG S6 [file mbio.02650-21-sf006.tif]
