# Supplementary material for: Contrasting Modes of New World Arenavirus Neutralization by Immunization-Elicited Monoclonal Antibodies
Source: mBio. 2022 Mar 22;13(2):e02650-21. doi: 10.1128/mbio.02650-21 (PMC9040744; doi:10.1128/mbio.02650-21)
Supplement: TABLE S1 [file mbio.02650-21-st001.docx]

**Table S1.** Summary for structurally characterized anti-JUNV and anti-MACV nAbs.

| **nAb** | **Specificity** | **Origin** | **Germline** | **Tyr211^hTfR1^-Mimicking Tyrosine** |  |
| --- | --- | --- | --- | --- | --- |
| GD01  (5EN2) | JUNV | Mouse | VK6-13*01 VH1-87*01 | CDR H3−Tyr98 | (*28, 34*) |
| OD01  (5NUZ) |  | Mouse | VK3-2*01 VH1-5*01 | CDR L1−Tyr30B | (*27, 34*) |
| JUN1  (7QU2) |  | Mouse | VK6-23*01 VH13-2*01 | CDR H3−Tyr113 |  |
| CR1-28  (5W1K) | JUNV  (reacts weakly with MACV) | Human | VK1-5*01 VH3-33*01 | CDR H3−Tyr106 | (*29*) |
| MAC1  (7QU1) | MACV | Mouse | VK10-96*01 VH1-80*01 | n/a |  |
| CR1-07  (5W1M) | MACV/JUNV | Human | VK4-1*01 VH3-30*15 | n/a | (*29*) |
