# Supplementary material for: Contrasting Modes of New World Arenavirus Neutralization by Immunization-Elicited Monoclonal Antibodies
Source: mBio. 2022 Mar 22;13(2):e02650-21. doi: 10.1128/mbio.02650-21 (PMC9040744; doi:10.1128/mbio.02650-21)
Supplement: TABLE S2 [file mbio.02650-21-st002.docx]

**Table S2.** Summary of anti-JUNV and anti-MACV nAb heavy and light chain features.

|  | **Heavy chain** | **V/D/J germline** | | | **Sequence identity*** | | **CDR sequence** | | | **CDR H3 length** |
| --- | --- | --- | --- | --- | --- | --- | --- | --- | --- | --- |
|  |  | **V** | **J** | **D** | **%V** | **%J** | **CDR H1** | **CDR H2** | **CDR H3** |  |
| Group 1 | JUN1_H | IGHV13-2*01 F | IGHJ4*01 F | IGHD2-3*01 F | 97.3 | 85.2 | GFTFSNYQ | ITVKSDNYGA | SRSGIYDGYYAYAMDY | 16 |
| Group 2 | JUN2_H | IGHV1-52*01 F | IGHJ4*01 F | IGHD3-3*01 F | 96.9 | 87.0 | GYTFTSYW | IDPSDSET | ARWGRLGVYFYTLDY | 15 |
|  | JUN3_H | IGHV1-52*01 F | IGHJ4*01 F | IGHD3-3*01 F | 94.8 | 87.0 | GYTFTSYW | IDPSDSET | ARWGRLGVYFYTLDY | 15 |
|  | JUN4_H | IGHV1-52*01 F | IGHJ4*01 F | IGHD3-3*01 F | 96.5 | 85.2 | GYTFTSYW | IDPSDSET | ARWGRLGVYFYTLDY | 15 |
|  | JUN5_H | IGHV1-52*01 F | IGHJ4*01 F | IGHD3-3*01 F | 96.5 | 85.2 | GYTFTSYW | IDPSDSET | ARWGRLGVYFYTLDY | 15 |
| Group 3 | JUN6_H | IGHV1-52*01 F | IGHJ2*01 F | IGHD3-1*01 F | 96.2 | 91.7 | GYTFTIYW | IDPSDSET | ARRTSSRGDYFDY | 13 |
|  | JUN7_H | IGHV1-52*01 F | IGHJ2*01 F | IGHD3-1*01 F | 97.2 | 91.7 | GYTFTSYW | IDPSDSET | ARRTSSRGDYFDY | 13 |
|  | MAC1_H | IGHV1-80*01 F | IGHJ2*01 F | IGHD1-1*01 F | 97.9 | 91.7 | GYAFGSHW | IYPGDGDT | ARDDYGTRYYFDY | 13 |
|  |  |  |  |  |  |  |  |  |  |  |
|  | **Kappa (light) chain** | **V/J germline** | | | **Sequence identity*** | | **CDR sequence** | | | **CDR L3 length** |
|  |  | **V** | **J** |  | **%V** | **%J** | **CDR L1** | **CDR L2** | **CDR L3** |  |
| Group 1 | JUN1_K | IGKV6-23*01 F | IGKJ5*01 F |  | 92.5 | 91.4 | QIVGTS | WAS | QQYATYPLT | 9 |
| Group 2 | JUN2_K | IGKV14-111*01 F | IGKJ1*01 F |  | 97.1 | 97.2 | QDINTY | RAN | LQHDEFPRA | 9 |
|  | JUN3_K | IGKV14-111*01 F | IGKJ1*01 F |  | 96.4 | 88.9 | QDINTY | RAN | LQHDEFPRT | 9 |
|  | JUN4_K | IGKV14-111*01 F | IGKJ1*01 F |  | 93.9 | 100.0 | QDINTY | RAN | LQHDEFPRT | 9 |
|  | JUN5_K | IGKV14-111*01 F | IGKJ1*01 F |  | 97.1 | 91.7 | QDINTY | RAN | LQHDEFPRT | 9 |
| Group 3 | JUN6_K | IGKV3-2*01 F | IGKJ1*01 F |  | 97.6 | 91.4 | ESLDNYGISF | TAS | QQNKEVPPT | 9 |
|  | JUN7_K | IGKV3-2*01 F | IGKJ1*01 F |  | 97.6 | 100.0 | ESVDNYGISF | TAS | QQSKEVPPT | 9 |
|  | MAC1_K | IGKV10-96*01 F | IGKJ1*01 F |  | 95.0 | 86.1 | QDINNY | YTS | QQGKTLPLTF | 10 |

*Nucleotide sequence identity between nAb sequence with native germline sequence.
