# Supplementary material for: Contrasting Modes of New World Arenavirus Neutralization by Immunization-Elicited Monoclonal Antibodies
Source: mBio. 2022 Mar 22;13(2):e02650-21. doi: 10.1128/mbio.02650-21 (PMC9040744; doi:10.1128/mbio.02650-21)
Supplement: TABLE S3 [file mbio.02650-21-st003.docx]

**Table S3.** Crystallographic data collection and refinement statistics.

| **Data Collection Statistics** | **JUNV GP1−Fab JUN1** | **MACV GP1−Fab MAC1** |
| --- | --- | --- |
| Beamline | DLS I03 | DLS I04 |
| Wavelength (Å) | 0.96859 | 0.97951 |
| Space Group | *P*2_1_ | *I*222 |
| Cell dimensions  *a, b, c* (Å) | 69.8, 66.8, 73.1 | 91.7, 100.7, 164.8 |
| 𝛼, β, 𝛾 (°) | 90, 103.5, 90 | 90, 90, 90 |
| Resolution range (Å) | 67.92−2.50 (2.54−2.50)* | 50.37−1.91 (1.94−1.91) |
| Rmerge | 0.131 (0.794) | 0.118 (>1) |
| I/σ (I) | 7.1 (1.4) | 7.3 (1.0) |
| CC_1/2_ | 0.995 (0.742) | 0.998 (0.571) |
| Completeness (%) | 99.8 (96.2) | 100 (99.4) |
| Multiplicity | 6.5 (5.9) | 6.8 (6.6) |
| **Refinement Statistics** |  |  |
| Resolution (Å) | 67.92−2.50 | 50.37−1.91 |
| No. reflections | 22,798 | 59,314 |
| *R_work_/R_free_* | 0.223/0.254 | 0.196/0.229 |
| **No. atoms**  Protein  Ligand  Water | 4,575  54  37 | 4,584  207  358 |
|  |  |  |
| **Average B-factors**  Protein  Ligand  Water | 74.9  93.7  69.7 | 43.9  67.6  45.2 |
|  |  |  |
| **Ramachandran (%)**  Favored  Allowed  Outlier | 96.9  3.1  0 | 97.6  2.4  0 |
|  |  |  |
| **Root mean square deviations (RMSD)**  Bond lengths (Å)  Bond angles (°) | 0.002  0.499 | 0.006  0.818 |
|  |  |  |

*The value for the highest-resolution shell is shown in parentheses.
